# Manganese affects the growth and metabolism of *Ganoderma lucidum* based on LC-MS analysis

**DOI:** 10.7717/peerj.6846

**Published:** 2019-05-01

**Authors:** Bo Zhang, Jie Zhou, Qiang Li, Bingcheng Gan, Weihong Peng, Xiaoping Zhang, Wei Tan, Lin Jiang, Xiaolin Li

**Affiliations:** 1 Soil and Fertilizer Institute, Sichuan Academy of Agricultural Sciences, Chengdu, China; 2 College of Life Science, Sichuan University, Chengdu, China; 3 Department of Microbiology, College of Resources, Sichuan Agricultural University, Chengdu, China

**Keywords:** *Ganoderma lucidum*, Manganese, Growth, Metabolism, LC-MS method

## Abstract

**Background:**

As a metal-enriched edible fungus, *Ganoderma lucidum* is capable of adsorbing manganese effectively. And the manganese ion is demonstrated to play an important role in the synthesis of manganese peroxidase (Mnp) and other physiological activities during *G. lucidum* growth. Recently, the influence of manganese on the metabolites of *G. lucidum* fruiting bodies can be revealed through metabonomics technique.

**Methods:**

In this study, we uncovered the changes between the control and 200 mg/kg Mn-treated fruiting bodies with liquid chromatography coupled to mass spectrometry (LC-MS).

**Results:**

The mycelial growth rate, dry yield, Mnp activity , total polysaccharide content, triterpenoid content, and total manganese content in the mature fruiting bodies of *G. lucidum* changed between the control and different Mn-treated groups. Based on LC-MS method, a total of 16 significantly different metabolites were obtained and identified, among which, five presented significantly down-regulated and 11 up-regulated in Mn-treated samples. The metabolites chavicol and palmitoylethanolamide were particularly significantly up-regulated, and were found the strong promotion relationship. Dependent on the MetPA database, four KEGG pathways were detected and glycerophospholipid metabolism was most impacted, in which, choline was involved in.

**Discussion:**

The added manganese ion in the substrate enhanced Mnp activities, and consequently promoted the mycelial growth, yield , metabolites in the fruiting bodies including triterpenoids, total manganese, chavicol, etc. Our finding can provide a theoretical reference to regulation of manganese on the physiological metabolism of *G. lucidum*.

## Introduction

As a medicinal material for centuries in China, *Ganoderma lucidum* is demonstrated to strengthen human bodies, especially the extracted polysaccharides and triterpenoids from fruiting bodies and spores (Wang et al., 2002; Sakamoto et al., 2016). With gradual maturation of cultivation techniques, the cultivation area of *G. lucidum* is expanding (Boh et al., 2007). *G. lucidum* growth is considered to be influenced by many factors (Stajic et al., 2002; Tanaka et al., 2016), among which, temperature is a major one. As is reported, the hyphal growth and extracellular enzyme activities are changed by temperature during fungal growth (Tommerup, 1983; Ni, Guo & Feng, 2001). Moreover, the physicochemical properties, associated with the bacterial composition in the substrate are thought to drive physiology and metabolism of *G. lucidum* at different growth stages (Zhang et al., 2018).

Enzymes are significant to the growth of white-rot fungi, and *G. lucidum* produces extracellular enzymes including manganese peroxidase (Mnp), laccase, etc. to degrade lignin and cellulose (Nagai et al., 2002). Mnp is considered to be one of the best characterized extracellular enzymes, and contributes to the initial lignin decomposition. Hofrichter et al. (1999) demonstrated that lignin can be mineralized dependent on Mnp and organic acids. The oxidant Mn^3+^ is produced by Mnp, and leads to redox of lignin polymer in most species of fungi (Hatakka, 1994). The manganese ion is also revealed to enhance laccase formation (Galhaup & Haltrich, 2001). Mn^3+^ serves as a part of the core enzyme structure, as well as the diffusible oxidant that participates in the degradation of lignin and cellulose polymers during the wood rot fungal metabolic process, thus, the wood rot fungi are more Mn^2+^ tolerant and absorb this element during the growth (Kähkönen, Lankinen & Hatakka, 2008; Elissetche et al., 2006; Blanchette, 1984). Besides, Mn^2+^ can interfere with fungal glycogen metabolism, nucleotides metabolism, cell transport and cell movement by partly replacing Ca^2+^ in calmodulin and changing its conformation (Xiao & Zhang, 2003).

Metals including manganese are considered as of importance to fungal biological systems (Schroeder, 1965), and fungi (e.g., *Pleurotus ostreatus*, *Daedalea quercina*) are capable of accumulating these metals at different concentrations with their individual preferences (Favero, Costa & Massimino, 1991; Sanglimsuwan et al., 1993; Gabriel et al., 1996). Manganese is less toxic and popularly used in metal enrichment, especially for *G. lucidum* (Tham, Matsuhashi & Kume, 1999). *G. lucidum* can enrich manganese, transforming the inorganic forms into the organic ones for human intake (Cai & Zhang, 2011). And the edible manganese poses important physiological functions for human body, involved in the enzyme synthesis and activation, immune function maintenance, blood sugar regulation, etc. (Elder et al., 2006). Metals (e.g., manganese) at certain concentrations help edible fungi with mycelial growth and fruiting body production (Yang et al., 2017). And previous studies of interaction between metals and edible fungi focus on metal adsorption, fungal mycelial growth, and biomass (Li et al., 2011). However, the effect of metal supplements (e.g., manganese) on the fungal metabolites remains less reported (Wei et al., 2008).

Metabonomics is a technique to study the metabolic networks of biological systems by observing the metabolite changes after stimulation (Fiehn et al., 2000). As an independent technology, liquid chromatography coupled to mass spectrometry (LC-MS) has been widely applied in metabolite change assessment due to its accuracy and quickness in determination of various compounds (Vélëz, Glassbrook & Daub, 2007; Zhang et al., 2014; Xu et al., 2015). The method is employed in mushroom metabolite detection including *Agaricus bisporus* (O’Gorman, Barry-Ryan & Frias, 2011), *Lentinula edodes* (Mata et al., 2014), the cultivated and wild tubers (Jamil et al., 2018), etc. With using LC/MS technology, some substances in the cells (e.g., nucleoside analogs), even some growth factors like guanosine and useful enzymes could be also detected and identified (Qiu et al., 2018; Jia et al., 2017). Qiu et al. (2018) revealed increasing exogenous metabolites induced by high-temperature based on LC-MS, and almost all the exogenous metabolites contributed to mycelial growth promotion of *Trichoderma asperellum*. Longo et al. (2017) investigated different chemical compounds from *Tuber melanosporum* samples stored under different storage atmospheres, and revealed the significance of glutathione and adenine as freshness indicators.

In this study, some important indicators of *G. lucidum* including mycelial growth rate, yield, Mnp activity, and content of nutrient components (e.g., total polysaccharides, triterpenoids, total manganese in the mature fruiting bodies) were investigated to reveal the effects of manganese on *G. lucidum* growth. Furthermore, LC-MS technology was used to uncover the interaction between manganese and fungal metabolites.

## Materials and Methods

### Cultivation of *Ganoderma lucidum*

The *G. lucidum* cultivar named Chuan Yuanzhi No. 1 was provided by the Soil and Fertilizer Institute at Sichuan Academy of Agricultural Sciences, and the cultivar has been deposited in the China General Microbiological Culture Collection Center (CGMCC) with a strain number CGMCC 13174. The substrate consisted of cottonseed hull (90%), wheat bran (5%), corn flour (4%), and gypsum (1%), and all of the materials were fresh, dry, and unspoiled. Manganese sulfate (MnSO_4_) was to provide manganese ions for cultivation of *G. lucidum.* MnSO_4_ solution with different concentrations were added into the substrates and the substrates were finally kept the concentrations at 50, 100, 150, 200, 250, 300, and 350 mg/kg, respectively. The substrate without MnSO_4_ addition was as control and all the substrates maintained 65% in water content. After fully mixed, the substrate was put into the polypropylene bags (size: 17 × 20 × 0.005 cm) and autoclaved at 100 °C for 18 h. Then the bags were cooled to room temperature and placed in laminar flow cabinet for inoculation of *G. lucidum*. After inoculation, they were planted in the cultivation site at Zhaojia, Jintang, China (N 30°48′16.45″, E 104°35′48.79″). A total of 400 cultivation bags were for statistics of growth indicators of *G. lucidum* in this study, and 50 bags were in each treatment.

### Sampling and determination of six physiological indexes

The sampling of *G. lucidum* was done at hyphal stage and mature stage. After inoculation, the mycelia of *G. lucidum* began to germinate and the mycelial growth rates were investigated. When the mycelia of *G. lucidum* spread and subsequently filled the whole culture media, substrate materials with mycelia were collected and Mnp was determined by trace kit method (Suzhou Comin Biotechnology Co. Ltd., Suzhou, China) following the manufacturer’s instructions. When the spores appeared on the pileus surface and gradually covered the yellow edges, it was the mature stage and the fruiting bodies of *G. lucidum* were collected and dried, then yield was investigated. Besides, the total polysaccharides and triterpenoids in the mature fruiting bodies were determined by phenol-sulfuric acid method (Zhang, 1987) and ultraviolet–visible spectrophotometry method (Fu et al., 2008), respectively. The total manganese in the fruiting bodies was determined according to GB 5009.268-2016. SPSS 19.0 software was used for statistical analysis by ANOVA and LSD methods with the *p*-value < 0.05.

### Metabolic sampling and detection

At the mature stage of *G. lucidum*, disposable disinfected gloves, sterilized tweezers and knives were prepared for metabolic sampling. Six duplicate samples of fruiting bodies were taken at control group and the treatment with 200 mg/kg MnSO_4_ addition. No less than 100 mg of fruiting bodies per sample was collected for subsequent detection. The fresh samples were stored at −20 °C in 10 mL tubes before sent. A total of 100 mg tissues of *G. lucidum* were transferred into five mL centrifuge tubes with five steel balls in. They were placed into liquid nitrogen for 5 min and then put in the high flux organization grinding apparatus, 70 Hz for 1 min. Afterward, 1,000 μL of methanol (Wokai ltd, pre-cooled at −20 °C) was added in the tubes and vortexed for 30 s (Vortex Mixer, QL-866). The tubes were subsequently placed into an ultrasound machine at room temperature for 30 min and vortexed for 60 s with addition of 750 μL chloroform (pre-cooled at −20 °C; Wokai Ltd., Jinhua, China) and 800 μL deionized water (ddH_2_O) (Arium^®^ mini, 4 °C; Sartorius, Gottingen, Germany). Then they were centrifuged for 10 min at 4 °C at 12,000 rpm (H1650-W; Eppendorf, Hamburg, Germany) and one mL supernatant was transferred into a new centrifuge tube. The supernatant samples were blow-dried by vacuum concentration (53050; Eppendorf) and dissolved with 250 μL methanol aqueous solution (1:1, 4 °C), filtered with 0.22 μm membrane (0.22 μm PTFE; Jin Teng, Shenzhen, China). Finally, samples were ready for LC-MS detection (De Vos et al., 2007).

Chromatographic separation was accomplished in a Shimadzu LC-30A system equipped with an ACQUITYUPLC® HSS T3-column (150 × 2.1 mm, 1.8 µm; Waters, Milford, MA, USA) maintained at 40 °C. The temperature of the autosampler was 4 °C. Gradient elution of analytes was carried out with 0.1% formic acid in water (A) (TCI) and acetonitrile (B) (Merck, Kenilworth, NJ, USA) at a flow rate of 0.3 mL/min. Injection of five μL of each sample was done after equilibration. An increasing linear gradient of solvent B (v/v) was used as follows: 0–0.5 min, 2% B; 0.5–9 min, 2–50% B; 9–12 min, 50–98% B; 12–13 min, 98% B; 13–14 min, 98–2% B; 14–15 min, 2% (Sangster et al., 2006). The ESI-MSn experiments were executed on the AB 5600+ mass spectrometer with the spray voltage of 5.50 and −4.50 kV in positive and negative modes, respectively. Gas 1 and gas 2 were both set at 50 psi, and curtain gas was 35 psi, and the source temperature was 500 °C. The mass analyzer scanned over a mass range of m/z 100–1,500 for full scan at the collision energy of 45 eV. Dynamic exclusion was implemented (Hillewaert et al., 2015).

### Data processing and analysis

Raw data were firstly converted to mzXML files by Proteowizard (v3.0.8789) (Smith et al., 2006). Then peaks identification, filtration, and alignment were done by XCMS package in R environment including major parameters bw = 5, ppm = 15, peakwidth = c(10,120), mzwid = 0.015, mzdiff = 0.01, and method = “centWave” and data matrices were subsequently obtained including mass to charge ratio (m/z), retention time, intensity, etc. Finally, a total of 25,107 precursor molecules were obtained by positive ion mode and 21,139 by negative ion mode. Subsequent analysis was carried on with data exported to excel. Batch normalization was done for comparison of data in different magnitudes. The metabolites of *G. lucidum* in different treatments were studied by LC-MS. After data preprocessing, multivariate statistical analyses, including principal component analysis, partial least squares analysis were taken to reveal the differences of metabolic compositions between groups. In addition, the relationships between identified metabolites and samples were uncovered by hierarchical clustering method and correlation analysis. Furthermore, KEGG pathways were used to analyze the biological significance of metabolites.

## Results

### Effects of manganese ion on *Ganoderma lucidum* growth

Six physiological indexes of *G. lucidum* (e.g., mycelial growth rate, dry yield, Mnp activity, total polysaccharide content, triterpenoid content, and total manganese content in the mature fruiting bodies) were determined in our study (Table 1). Obviously, *G. lucidum* growth was affected by manganese ion additive in the substrate, and all the tested physiological indexes of *G. lucidum* changed with different manganese ion concentrations. The mycelia grew slowest without manganese ion addition and it was significantly different from Mn-treated samples. The highest mycelial growth rate was found in the treatment with addition of 100 mg/kg Mn, reaching 6.18 mm/d. The investigation revealed the promotion of added manganese on *G. lucidum* yield and the treatment with addition of 350 mg/kg Mn exhibited the maximum yield (42.60 g per bag on average), 56.04% higher than the control. Moreover, Mnp was more active in the Mn-treated samples and it roughly performed a first rise and then a fall with the increase of manganese ion concentration. The highest Mnp activity (82.40 nmol/min/g on average) was found in the treatment with the manganese concentration of 200 mg/kg. It was 2.69 folds as high as that of the control. Besides, triterpenoid content and total manganese content in *G. lucidum* fruiting bodies were both increased through adding manganese in the substrate. However, total polysaccharide contents in mature fruiting bodies was decreased, and the richest polysaccharide content was detected in the control (2.13%), followed by the treatment with addition of 50 mg/kg Mn (2.11%) and both were significantly different from that of other treatments.

**Table 1:** The tested physiological indexes of *Ganoderma lucidum* in different treatments.

NO.	MGR (mm/d)	Yield (g)	MnP (nmol/min/g)	TP (%)	TT (%)	TM (mg/kg)
CK	5.70 ± 0.19^b^	27.30 ± 1.79^d^	30.65 ± 0.32^f^	2.13 ± 0.12^a^	2.46 ± 1.32^a^	7.20 ± 0.30^e^
Mn50	6.15 ± 0.15^a^	34.00 ± 2.70^bcd^	46.87 ± 1.14^e^	2.11 ± 0.30^a^	3.71 ± 0.37^a^	11.97 ± 0.06^c^
Mn100	6.18 ± 0.21^a^	32.10 ± 2.20^bcd^	60.38 ± 0.41^c^	1.13 ± 0.18^b^	3.17 ± 1.72^a^	10.30 ± 0.10^d^
Mn150	6.15 ± 0.09^a^	36.60 ± 1.94^ab^	67.83 ± 1.29^b^	1.28 ± 0.03^b^	3.35 ± 0.51^a^	10.43 ± 0.12^d^
Mn200	5.95 ± 0.12^ab^	27.90 ± 2.98^cd^	82.40 ± 1.33^a^	1.19 ± 0.17^b^	3.57 ± 0.24^a^	13.13 ± 0.15^b^
Mn250	6.00 ± 0.07^ab^	35.20 ± 1.85^bc^	52.69 ± 1.71^d^	1.44 ± 0.48^b^	3.05 ± 0.32^a^	13.30 ± 0.00^b^
Mn300	5.85 ± 0.09^ab^	38.80 ± 1.26^ab^	56.83 ± 1.73^cd^	1.21 ± 0.27^b^	2.64 ± 0.80^a^	13.00 ± 0.50^b^
Mn350	5.98 ± 0.11^ab^	42.60 ± 2.95^a^	71.37 ± 2.28^b^	1.21 ± 0.04^b^	2.95 ± 0.45^a^	15.37 ± 0.45^a^

**Notes:**

NO., substrates with different manganese ion concentration; MGR, mycelial growth rate; Yield, the dry yield per bag; MnP, the activity of manganese peroxidase determined with fresh weight; TP, the total content of polysaccharides in mature fruiting bodies; TT, the content of triterpenoids in mature fruiting bodies; TM, the total content of manganese in mature fruiting bodies. CK, the control group without MnSO_4_ addition; Mn50, the treatment group with 50 mg/kg MnSO_4_ addition; Mn100, the treatment group with 100 mg/kg MnSO_4_ addition; Mn150, the treatment group with 150 mg/kg MnSO_4_ addition; Mn200, the treatment group with 200 mg/kg MnSO_4_ addition; Mn250, the treatment group with 250 mg/kg MnSO_4_ addition; Mn300, the treatment group with 300 mg/kg MnSO_4_ addition; Mn350, the treatment group with 350 mg/kg MnSO_4_ addition.

Data with different lower-case letters display significant differences (*p*-value < 0.05) by LSD method of one-way ANOVA. MGR, Yield, MnP, and TP are with more than three replicates.

### Untargeted metabolomics analysis

**Figure 1:**
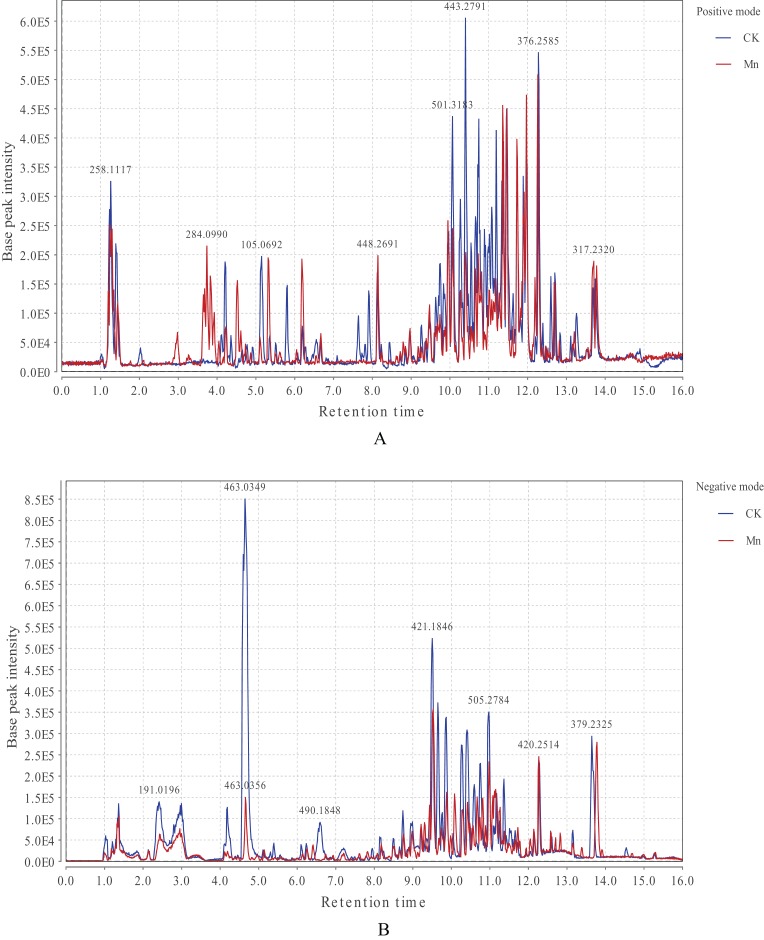
LC-MS base peak chromatograms of CK (in blue) and Mn (in red) samples in positive (A) and negative ionization mode (B). CK, the control group without MnSO_4_ addition; Mn, the treatment group with 200 mg/kg MnSO_4_ addition. *X*-axis represents the retention time, and *Y*-axis represents base peak intensity. The figure displays the detected peaks in positive (A) and negative (B) ionization modes. The strongly detected molecules of metabolites in *G. lucidum* fruiting bodies exhibited a decrease or an increase between the control and the Mn-treated samples.

The LC-MS base peak chromatograms of *G. lucidum* extracts were displayed in Fig. 1. A total of 25,107 peaks were detected in positive mode and 21,139 in negative ionization after the xcms preprocessing of the original mass spectrometry data. Some specific metabolites in *G. lucidum* fruiting bodies exhibited a decrease or an increase compared with the control and the Mn-treated samples. In detail, relative intensity of ionic strength in positive ionization mode showed a higher level during the retention time of 3.0–4.0 min in 200 mg/kg Mn-treated fruiting bodies, and a molecule with m/z 284.10 increased obviously at the retention time of 225 s. In addition, it performed a higher level in Mn-treated fruiting bodies at the retention time of 271 s, 319 s, and 371 s. Nevertheless, most of molecules were strongly detected in the control group, in which a molecule with m/z 443.28 showed the highest intensity of ionic strength at 624 s. What’s more, the control samples exhibited an absolute high level in relative intensity of ionic strength in negative ionization mode, and a molecule with m/z 463.03 showed the strongest ionic intensity. Instead, two molecules with m/z 420.25 and m/z 379.23 in Mn-treated fruiting bodies were with stronger ionic intensity at the retention time of 736 s, 827 s, respectively.

### PLS-DA analysis

**Figure 2:**
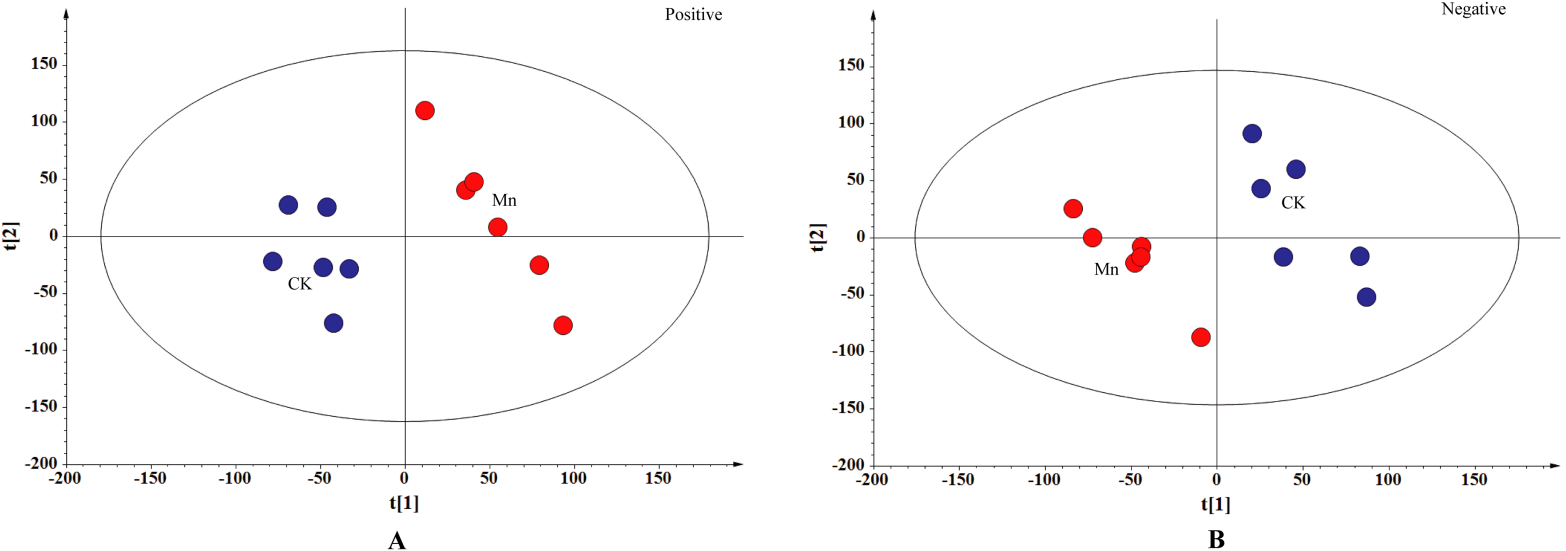
PLS-DA score plot. CK, the control group without MnSO_4_ addition; Mn, the treatment group with 200 mg/kg MnSO_4_ addition. The control samples were in blue and the Mn-treated samples in red. (A) Positive ionization mode, R2X[1] = 0.157, R2X[2] = 0.156, Ellipse: Hotelling’s T2 (95%); (B) negative ionization mode, R2X[1] = 0.183, R2X[2] = 0.136, Ellipse: Hotelling’s T2 (95%). PLS-DA analysis is to distinguish the different metabolites between the Mn-treated and control samples of *G. lucidum*.

**Figure 3:**
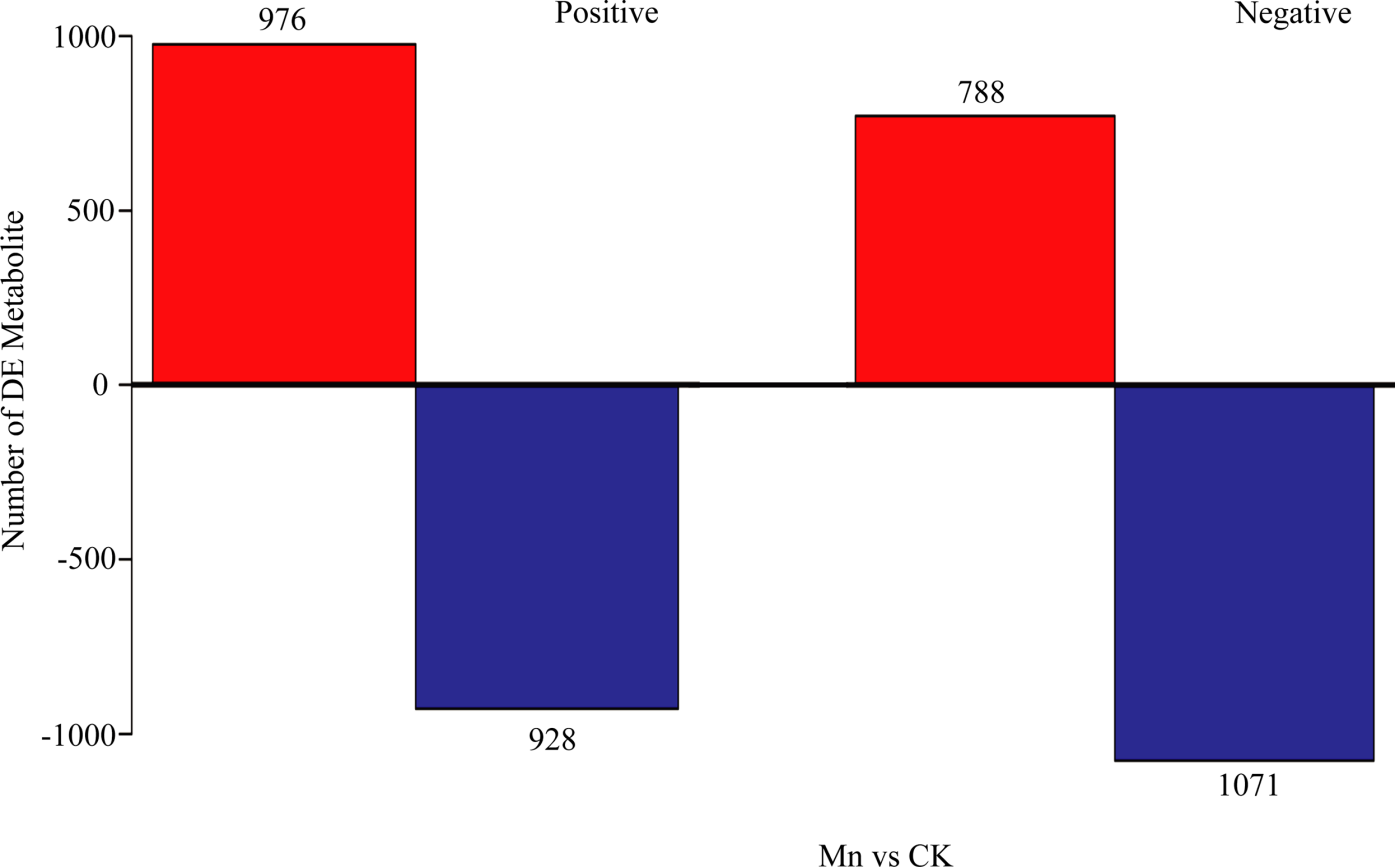
Investigation of differential metabolites comparing the Mn-treated samples to the control. CK, the control group without MnSO_4_ addition; Mn, the treatment group with 200 mg/kg MnSO_4_ addition. *X*-axis represents the samples, and *Y*-axis represents number of DE metabolites. The up-regulated metabolites are in red, and the down-regulated are in blue.

To distinguish the different metabolites between the Mn-treated and control samples of *G. lucidum*, the metabolites of the two treatments were compared in both positive and negative ionization mode using PLS-DA analysis (Fig. 2). The samples with 200 mg/kg manganese ion treated were obviously separated from the control in both ionization modes based on the interpretable degree 0.313, 0.319 in positive and negative ionization mode, respectively. The results revealed a prominent difference of the LC-MS produced metabolites in fruiting bodies of *G. lucidum* between the Mn-treated and control samples. Furthermore, a total of 976 metabolites were up-regulated and 928 down-regulated in the Mn-treated samples in positive ionization mode. While there were 788 up-regulated and 1,071 down-regulated metabolites detected in the Mn-treated samples in negative ionization mode (Fig. 3).

### Screening of differential metabolites

**Figure 4:**
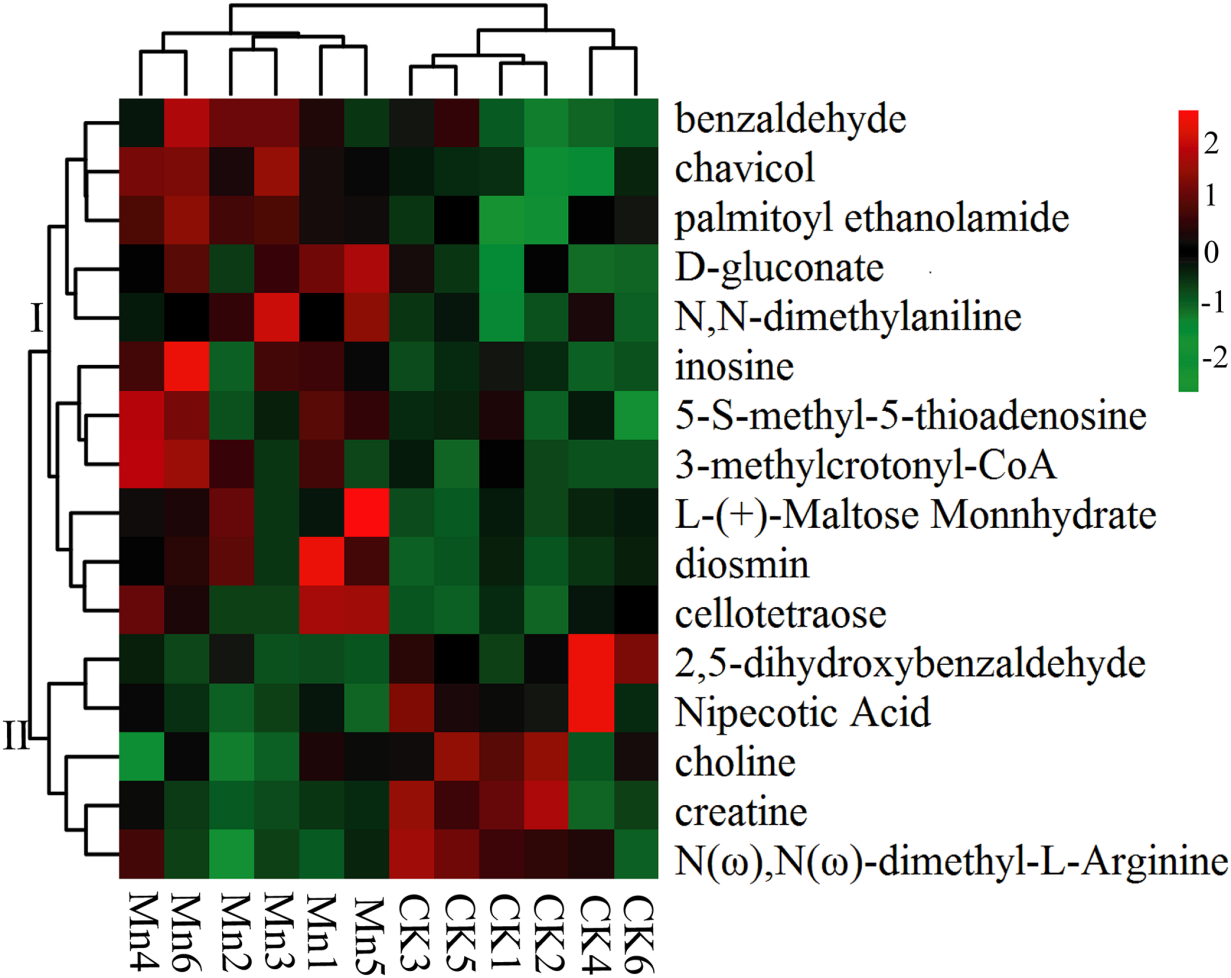
Heat map of significantly differential metabolites. CK, the control group without MnSO_4_ addition; Mn, the treatment group with 200 mg/kg MnSO_4_ addition. There are six duplications in each treatment. The samples were subject to bidirectional clustering analysis using the R package Pheatmap based on the euclidean distance and complete linkage clustering. The up-expressed metabolites are colored in red and the down-regulated metabolites in green, respectively. The heat map presents and groups all the significantly differential metabolites.

The main metabolites were fragmented and screened and putatively identified. A total of 16 significantly different metabolites were obtained by accurate molecular weight confirmation and annotation to Human Metabolome Database, Metlin, massbank, LipidMaps, and mzclound according to the MS/MS fragmentation mode (Table 2). The identified metabolites mainly included organic acids, aldehydes, and glycosides. Among them, five metabolites presented significantly down-regulated in Mn-treated samples including 2,5-dihydroxybenzaldehyde, N(ω), N(ω)-dimethyl-L-arginine, creatine, choline and nipecotic acid, and the other 11 were significantly up-regulated. The results were also presented in a heat map (Fig. 4). All the significantly differential metabolites were divided into two groups. The up-expressed metabolites of Mn-treated *G. lucidum* were in group I, while the down-expressed metabolites were clustered in group II. Particularly, chavicol and palmitoylethanolamide were extremely significantly up-regulated (*p*-value < 0.01) (Fig. S1). For normalized intensity, chavicol of Mn-treated samples was 61.77% higher than that of the control (VIP = 2.23, *p*-value = 0.0013). More than that, palmitoylethanolamide of Mn-treated samples was 2.01 folds as high as that of the control (VIP = 2.04, *p*-value = 0.0059).

**Table 2:** Significantly differential metabolites of *Ganoderma lucidum* between two treatments.

Putative identification	m/z	rt (s)	RMM	MF	PT	log2fc(Mn/CK)	*p*-value
Chavicol	135.08	775.06	134.18	C_9_H_10_O	[M+H]^+^	0.69	0.0013
Palmitoylethanolamide	300.29	882.81	299.49	C_18_H_37_NO_2_	[M+H]^+^	1.01	0.0059
D-gluconate	195.05	101.26	196.06	C_6_H_12_O_7_	[M−H]^−^	0.92	0.0126
Diosmin	610.17	358.73	608.54	C_28_H_32_O_15_	[M+H]^+^	1.86	0.0142
*N*,*N*-Dimethylaniline	122.10	769.89	121.18	C_8_H_11_N	[M+H]^+^	0.95	0.0208
Benzaldehyde	107.05	775.05	106.12	C_7_H_6_O	[M+H]^+^	0.68	0.0233
2,5-Dihydroxybenzaldehyde	137.02	428.47	138.12	C_7_H_6_O_3_	[M−H]^−^	−2.29	0.0248
Inosine	267.07	254.89	268.23	C_10_H_12_N_4_O_5_	[M−H]^−^	2.02	0.0277
3-Methylcrotonyl-CoA	851.40	743.50	849.63	–	[M+H]^+^	1.71	0.0310
Cellotetraose	667.23	82.59	666.58	C_24_H_42_O_21_	[M+H]^+^	1.61	0.0313
N(ω),N(ω)-dimethyl-L-arginine	203.15	84.51	202.25	C_8_H_18_N_4_O_2_	[M+H]^+^	−0.80	0.0347
L-(+)-Maltose monnhydrate	360.15	106.87	360.31	–	[M+H]^+^	1.35	0.0394
Creatine	261.13	358.99	131.13	C_4_H_9_N_3_O_2_	[M−H]^−^	−1.05	0.0423
Choline	104.11	236.68	103.10	C_5_H_13_NO	[M+H]^+^	−1.10	0.0430
5-S-methyl-5-thioadenosine	298.10	310.62	297.33	C_11_H_15_N_5_O_3_S	[M+H]^+^	0.85	0.0431
Nipecotic acid	130.09	65.12	129.08	C_6_H_11_NO_2_	[M+H]^+^	−0.83	0.0490

**Note:**

m/z, mass charge ratio; rt, retention time; RMM, relative molecular mass; MF, molecular formula; PT, precursor type.

### Correlation and pathway analysis of differential metabolites

**Figure 5:**
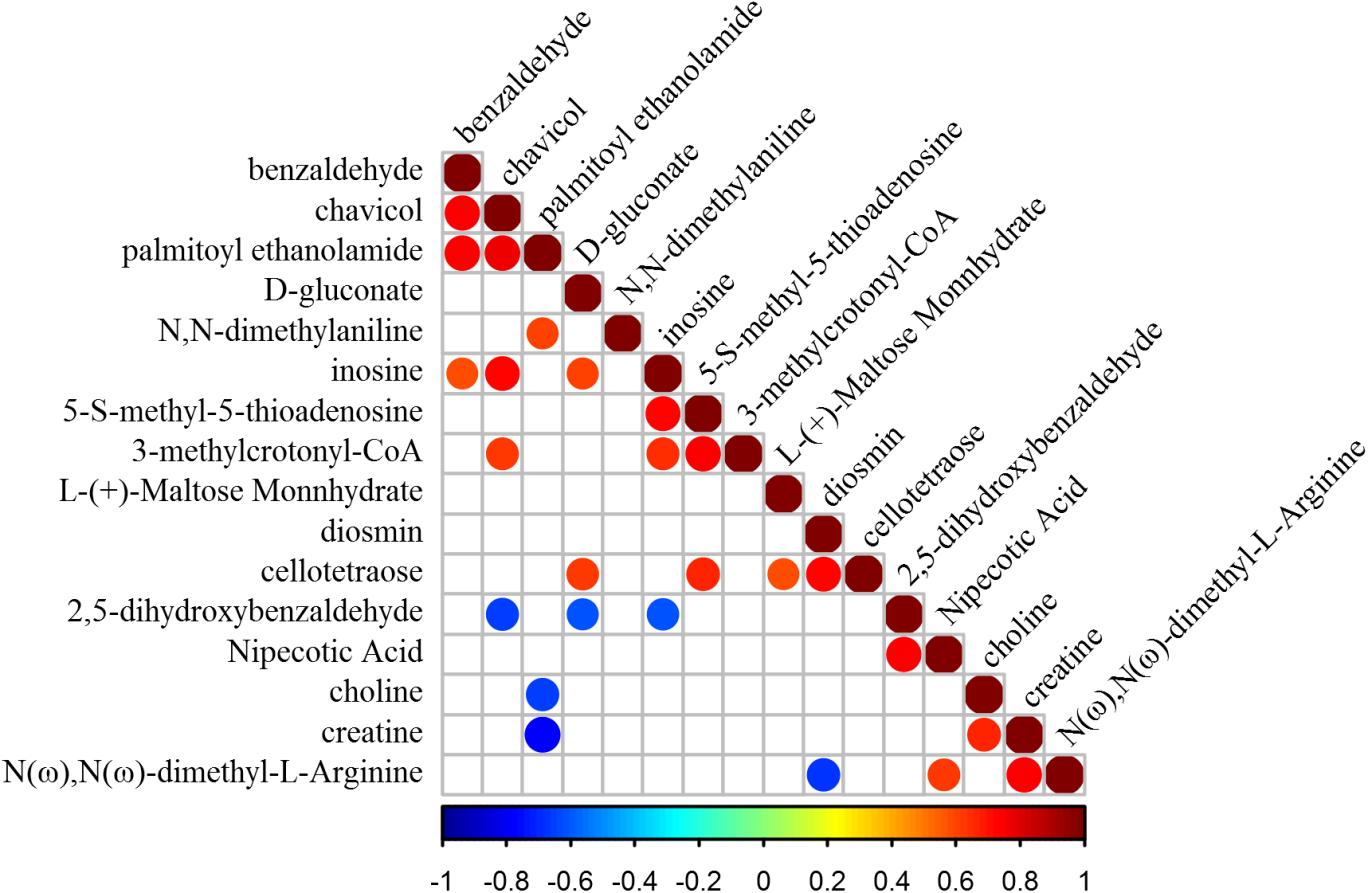
Correlation heat map of differential metabolites. The blank squares showed no relevance between differential metabolites based on the statistical test (*p*-value > 0.05). The squares with color markings showed mutual promotion or inhibition relationships between differential metabolites (*p*-value < 0.05).

Mutual promotion or inhibition relationships between differential metabolites were exhibited using correlation analysis (*p*-value < 0.05) (Fig. 5). Most of the differential metabolites between two treatments were without relevance (e.g., chavicol and D-gluconate, cellotetraose and nipecotic acid). Some were obviously correlated with other metabolites. Inosine presented a positive correlation with five metabolites (e.g., 3-methylcrotonyl-CoA, 5-S-methyl-5-thioadenosine, chavicol, D-gluconate, and benzaldehyde), suggesting its extensive promotion effect on other metabolites. In particular, chavicol and palmitoylethanolamide promoted each other strongly with the highest correlation coefficient 0.76. Instead, another metabolite, 2,5-dihydroxybenzaldehyde, showed a negative correlation with three metabolites (e.g., chavicol, D-gluconate, inosine). What’s more, creatine and choline were mutually promoted, and both were inhibited by palmitoylethanolamide, revealing a possible similarity of them (e.g., element composition, structure, etc.). Besides, the metabolic pathways of differential metabolites between Mn-treated and control samples were uncovered according to the MetPA database. A total of four KEGG pathways (e.g., pentose phosphate pathway, glycerophospholipid metabolism, cysteine and methionine metabolism, and purine metabolism) were identified to be possibly biologically disturbed in this study (Table 3). Glycerophospholipid metabolism was the most impacted one (http://www.kegg.jp/pathway/sce00564+C00114). The metabolite annotated to the pathway of glycerophospholipid metabolism was choline (Fig. S2), which was down-regulated with Mn treated in the fruiting body of *G. lucidum*.

**Table 3:** Metabolic pathways information.

KEGG	Total	Expected	Hits	Raw *p*	−log(*p*)	FDR	Impact
Pentose phosphate pathway	18	0.27	1	0.24	1.42	1	0
Glycerophospholipid metabolism	26	0.39	1	0.33	1.10	1	0.0112
Cysteine and methionine metabolism	33	0.50	1	0.40	0.91	1	0.0108
Purine metabolism	60	0.91	1	0.61	0.49	1	0.0059

**Note:**

Total, the total number of metabolites in targeted metabolic pathway; Expected, one parameter; Hits, the actually matched number from the user uploaded data; Raw *p*, the original *p*-value calculated from the enrichment analysis; FDR, the *p*-value adjusted using False Discovery Rate; Impact, the pathway impact value calculated from pathway topology analysis.

## Discussion

The present study revealed an effective uptake of manganese ions by *G. lucidum*. With MnSO_4_ added in the substrate, the mycelia of *G. lucidum* increased in the growth rate, significantly faster than that of control group. As is reported, lower metal concentrations, especially Mn^2+^, are found to improve fungal growth rate (Falih, 1998). However, Mn^2+^ with a concentration over 500 mg/L was reported to significantly inhibit mycelial growth of *Pholiota adiposa* (Zhang et al., 2015). Meanwhile, the manganese promoted *G. lucidum* yield in present study, but excessive Mn^2+^ would lead to salt stress, resulting in biomass decrease, as well as yield reduction (Pan, 1995). According to Chiu et al. (1998), the yield of *Pleurotus ostreatus* fruiting bodies is decreased by manganese, probably due to the higher manganese concentration in the substrate.

Manganese peroxidase activity is closely related to the mycelial growth rate and yield of *G. lucidum*. In the present study, the Mnp activity exhibited a general elevation, increasing firstly and then decreasing with manganese added in the substrate. As an essential trace element, manganese is capable of activating intracellular enzyme reactions (e.g., hydrolysis, reduction, and phosphorylation) (Pan, 1995), and participating in the catalytic cycle of Mn-dependent peroxidase, involved in lignin degradation (Périé & Gold, 1991; Perez & Jeffries, 1992). Mnp is a directly manganese-affected enzyme and plays an important role in the whole life cycle of *G. lucidum* (Jellison et al., 1997). As is reported, the peroxidase and catalase in the mycelia of *Cordycep militaris* generally showed a first increase and a following decrease with the increasing concentration of manganese in the range of 10–100 g/L in the medium (Zuo, 2013), which was in accord with the conducted Mnp in the present study. However, the Mnp activity was decreased at the concentration of 250 mg/kg Mn in the substrate. Accordingly, the presence of metals (e.g., Mn^2+^) at certain concentrations would inhibit fungal enzymatic reactions (e.g., *Pleurotus chrysosporium*) by interfering with the carbon and energy supplying system (Falih, 1998). Thus, the manganese concentration at 250 mg/kg was revealed to potentially inhibit Mnp activity in the present study. Generally speaking, the supplemented manganese significantly accelerated the Mnp activity, which would aid to nutrient uptake by the studied *G. lucidum* from the substrate.

The metabolites in the *G. lucidum* fruiting body changed with the manganese addition in the present study. The most abundant polysaccharide content was found in the fruiting bodies without manganese addition, which was in disagreement with Zhang et al. (2015) study on *Pholiota adiposa*. The impact of manganese on fungal polysaccharide formation is multifaceted, and varies with different concentrations, mushroom species, and growth environments. The specific mechanism needs to be further explored. Moreover, the manganese reduced the LC-MS produced metabolites including creatine, etc., but promoted the other 11 metabolites, especially chavicol and palmitoylethanolamide. It was probably combined with the different mycelial growth rates and Mnp activities caused by the manganese addition. It’s demonstrated that the metabolites may be influenced by the growth characteristic, growth stage, and postharvest condition (Kalač, 2013), and environmental alteration like increased manganese concentration is also likely to impact fungal growth, and influence the secondary metabolites (Deduke, Timsina & Piercey-Normore, 2012). The essential metals (e.g., copper, manganese, zinc) enter the fungal cells, and tend to biologically influence the fungal metabolic process (Hughes & Poole, 1991). The direct performance is accumulation of the added mineral element. Our study also revealed a significant increase of manganese content in the *G. lucidum* fruiting bodies of each treatment with manganese addition compared with the control. When the fungal fruiting bodies communicate with the substrate by translocation, their cell walls play key roles of absorbing metals (Brunnert & Zadražil, 1979). With the metal accumulation, fungi tend to construct a metal bioremediation, and consequently undergo significant changes in metabolite production (e.g., exopolysaccharides, extracellular enzymes, organic acids) (Mishra & Malik, 2014).

A wide variety of secondary metabolites are produced during the growth process of the studied *G. lucidum*, and different fungal metabolites are with different properties and functions. Choline, detected in the present study, serves as a receptor molecule for nerve signal transduction, as well as one important component to maintain the stability and integrity of cell structure (Wang, 2016). This substance was decreased by manganese supplements probably because choline was consumed for synthesis of other substances during *G. lucidum* growth. Notably, chavicol and palmitoylethanolamide were significantly up-expressed in response to manganese supply. Chavicol belongs to phenolic compound with antioxidant and scavenging ability (Boonsong, Klaypradit & Wilaipun, 2016), and palmitoylethanolamide is considered to be anti-inflammatory (Hoareau et al., 2009). Moreover, the triterpenoid contents in the mature fruiting bodies of experimental groups remained a higher level than that of control group, while the manganese above a concentration of 50 mg/kg was to decrease polysaccharide formation. As is reported, polysaccharides and triterpenoid in *G. lucidum* are demonstrated to be anticancer and antibacteria, and β-glucans are particularly biologically active (Wasser, 2002). Accordingly, the supplemented manganese is capable of regulating active substances in *G. lucidum* fruiting bodies in the present study.

As stated above, manganese adsorption influenced metabolites of *G. lucidum*, however, different metabolites also impacted the manganese accumulation. One of the contributors to metal adsorption of *G. lucidum* is chitin (Muraleedharan & Venkobachar, 1990). In addition, the polysaccharides in fungal cell wall with active components (e.g., sulfhydryl group, carboxyl group, etc.) and elements (e.g., nitrogen, oxygen, phosphorus, and sulfur) are demonstrated to participate in metal adsorption in the environment (Zhang & Liu, 2002). Thus, manganese interacts with metabolites, and ultimately completes the normal growth of *G. lucidum*.

Metabonomics has been applied in the present study, and the accurate extraction and acquisition of metabolites aids to further analysis of cellular metabolic pathways (Doerfler et al., 2013). A total of four KEGG pathways were identified, and the dominant one was glycerophospholipid metabolism. As the most common phospholipids, glycerophospholipids are demonstrated to form biofilms and participate in protein recognition. The synthesis of glycerophospholipids consists of source process, activation process, and production process, in which choline is one of the sources, and is subsequently activated to CDP-choline (Gupta, Radhakrishnan & Khorana, 1977). As it happened, the identified metabolite choline was significantly down-expressed affected by manganese in the present study, which validated the use of choline in glycerophospholipid metabolism. Meanwhile, carbohydrates play a role of energy supply, and they are catabolized for energy production during the fungal growth (Barros et al., 2007). L-(+)-maltose monnhydrate, increased by manganese addition, was likely to provide energy for biological pathways of *G. lucidum*. Besides, two identified cerebrosides in the fruiting bodies of *G. lucidum* are reported to depress DNA polymerase of eukaryotic species (Zaidman et al., 2005; Gan et al., 1998). Accordingly, the up-regulated metabolite, 5-S-methyl-5-thioadenosine, was related to DNA synthesis pathway. In general, the metabolite changes caused by manganese similarly affected biological pathways of *G. lucidum*, and eventually promoted its growth.

## Conclusions

The present study uncovered an obvious effect of manganese ion additive on metabolites and physiological indicators of *G. lucidum*. The added manganese ion promoted Mnp activities, the mycelial growth, and dry yield of *G. lucidum*. The detected metabolites including triterpenoids, chavicol, palmitoylethanolamide, etc. were increased by manganese addition, suggesting a promotion of manganese on nutritional value of *G. lucidum*. Besides, these changed metabolites possessed mutual promotion or inhibition relationships, and were involved in biological pathways of *G. lucidum*, which also impacted manganese absorption. Furthermore, in the process of manganese intake by *G. lucidum*, choline was revealed to participate in the most impacted pathway, glycerophospholipid metabolism. Hence, our study approved that the supplemented manganese played a role of promoting the *G. lucidum* growth and its metabolites. And it would aid in manganese-enriched cultivation of *G. lucidum*.

## Supplemental Information

10.7717/peerj.6846/supp-1 Supplemental Information 1 Box-plot of two extremely significantly up-regulated metabolites (e.g., chavicol, palmitoylethanolamide) (*p*-value < 0.01). CK, the control group without MnSO_4_ addition; Mn, the treatment group with 200 mg/kg MnSO_4_ addition. *X*-axis represents the samples, and *Y*-axis represents the normalized intensity.

10.7717/peerj.6846/supp-2 Supplemental Information 2 The KEGG pathway of glycerophospholipid metabolism. The KEGG pathway of glycerophospholipid metabolism comes from http://www.kegg.jp/pathway/sce00564+C00114. The red circle was the hit metabolite annotated to the pathway of glycerophospholipid metabolism.

10.7717/peerj.6846/supp-3 Supplemental Information 3 Dataset S1. The raw data were for the LC-MS analysis including PLS-DA analysis in both positive and negative ionization mode, significantly differential metabolites of *Ganoderma lucidum* between treatments, mutual promotion or inhibition relationships between differential metabolites, etc.

## Additional Information and Declarations

### Data Availability

The following information was supplied regarding data availability: Raw data is available in the Supplemental Files.
