# Supplementary figures and images for: Manganese affects the growth and metabolism of Ganoderma lucidum based on LC-MS analysis
Source: PeerJ. 2019 May 1;7:e6846. doi: 10.7717/peerj.6846 (PMC6500383; doi:10.7717/peerj.6846)

**chavicol**

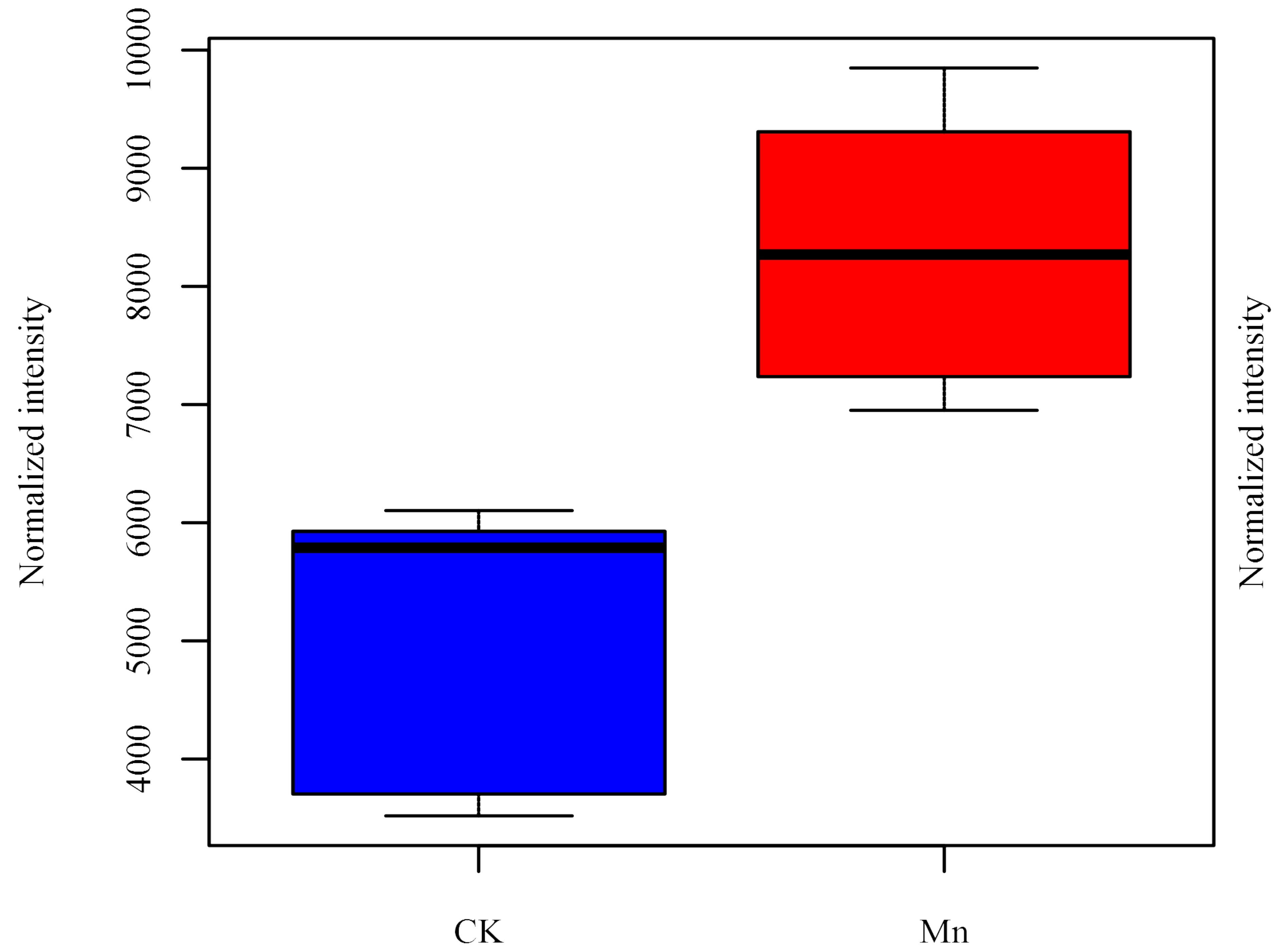

**palmitoyl ethanolamide**

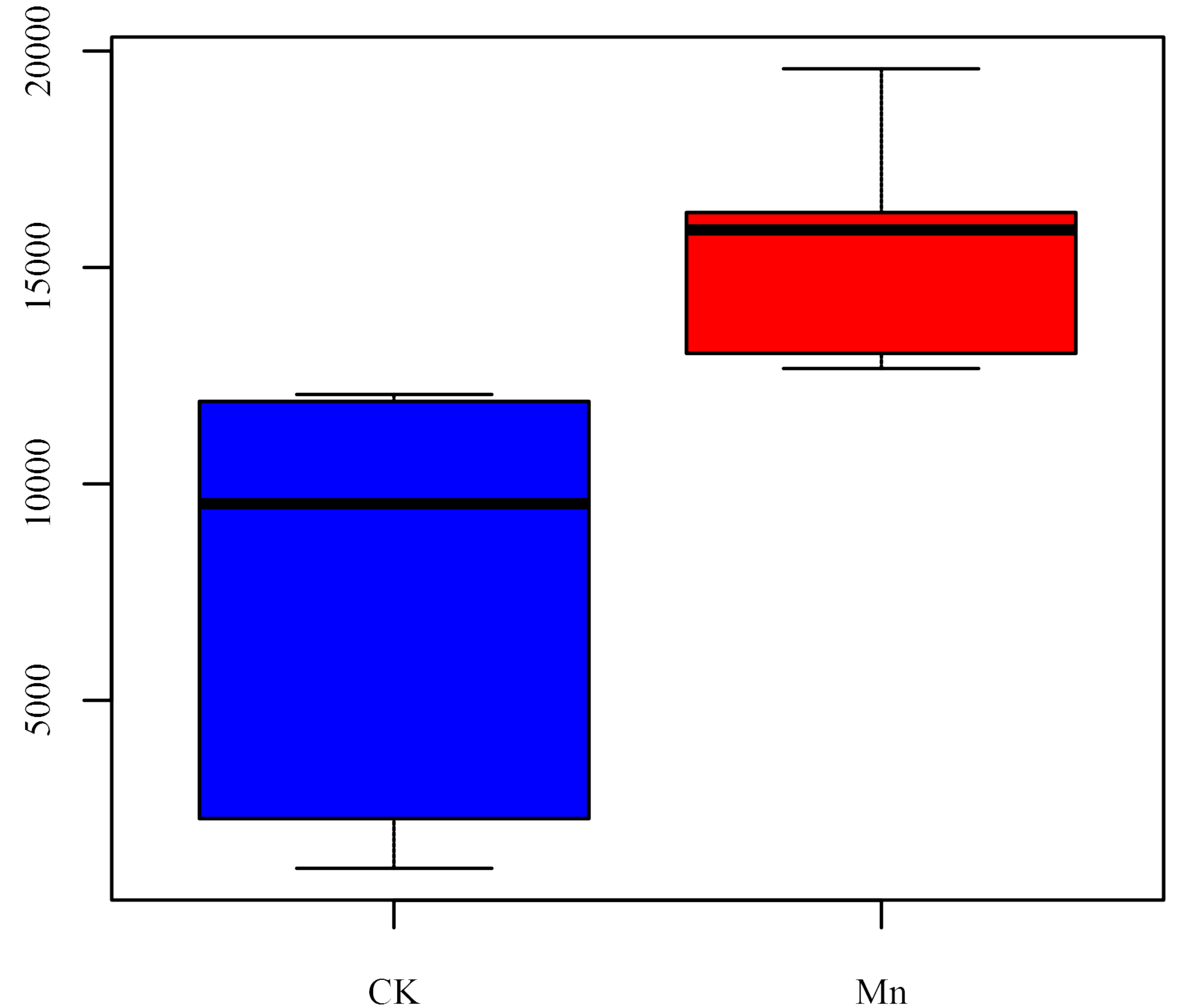

Supplement: Supplemental Information 1 — CK, the control group without MnSO4 addition; Mn, the treatment group with 200 mg/kg MnSO4 addition. X-axis represents the samples, and Y-axis represents the normalized intensity. [file peerj-07-6846-s001.pdf]

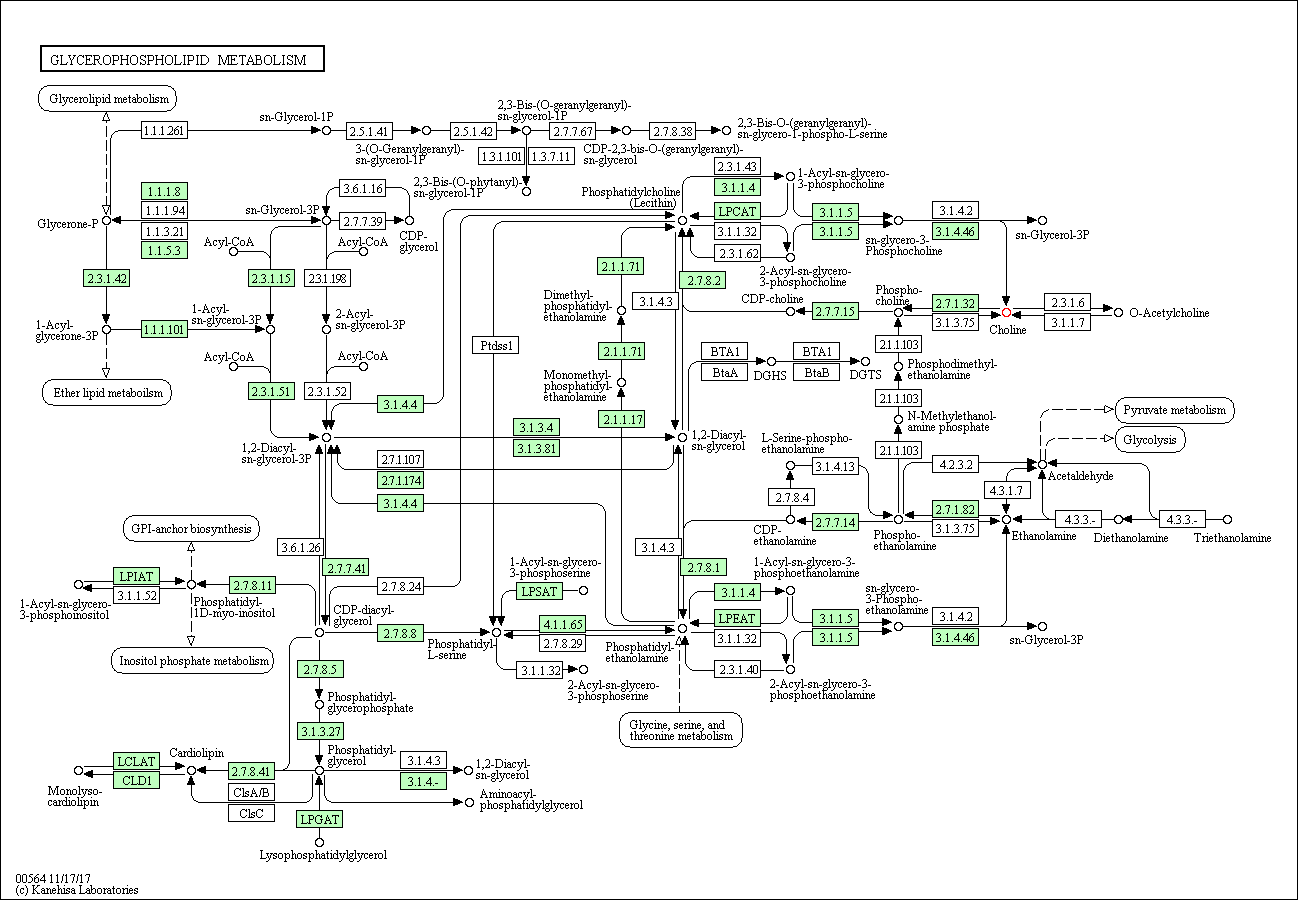

Supplement: Supplemental Information 2 — The KEGG pathway of glycerophospholipid metabolism comes from http://www.kegg.jp/pathway/sce00564+C00114. The red circle was the hit metabolite annotated to the pathway of glycerophospholipid metabolism. [file peerj-07-6846-s002.png]

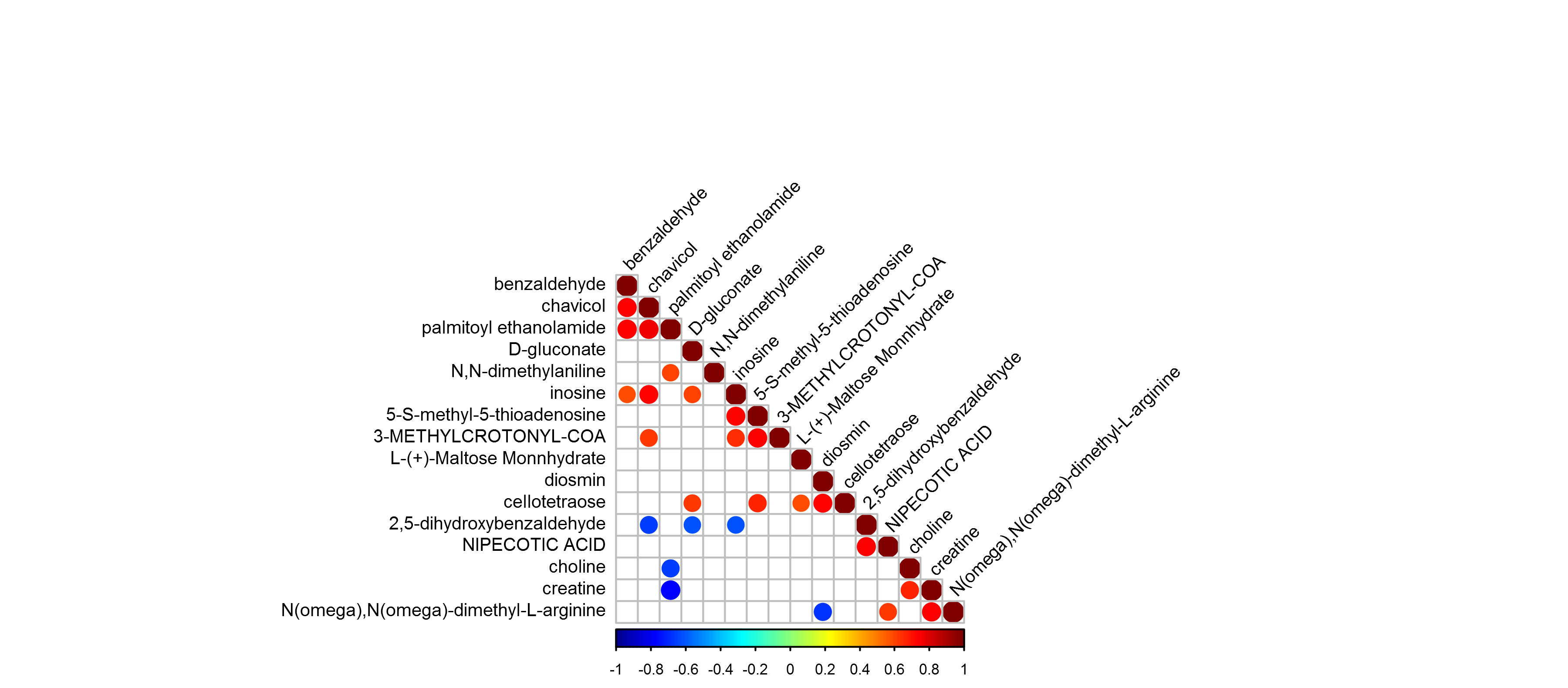

Supplement: Supplemental Information 3 — The raw data were for the LC-MS analysis including PLS-DA analysis in both positive and negative ionization mode, significantly differential metabolites of Ganoderma lucidum between treatments, mutual promotion or inhibition relationships between differential metabolites, etc. [file peerj-07-6846-s003.zip › raw data/CK vs Mn/cluster/correlation_dem.png]

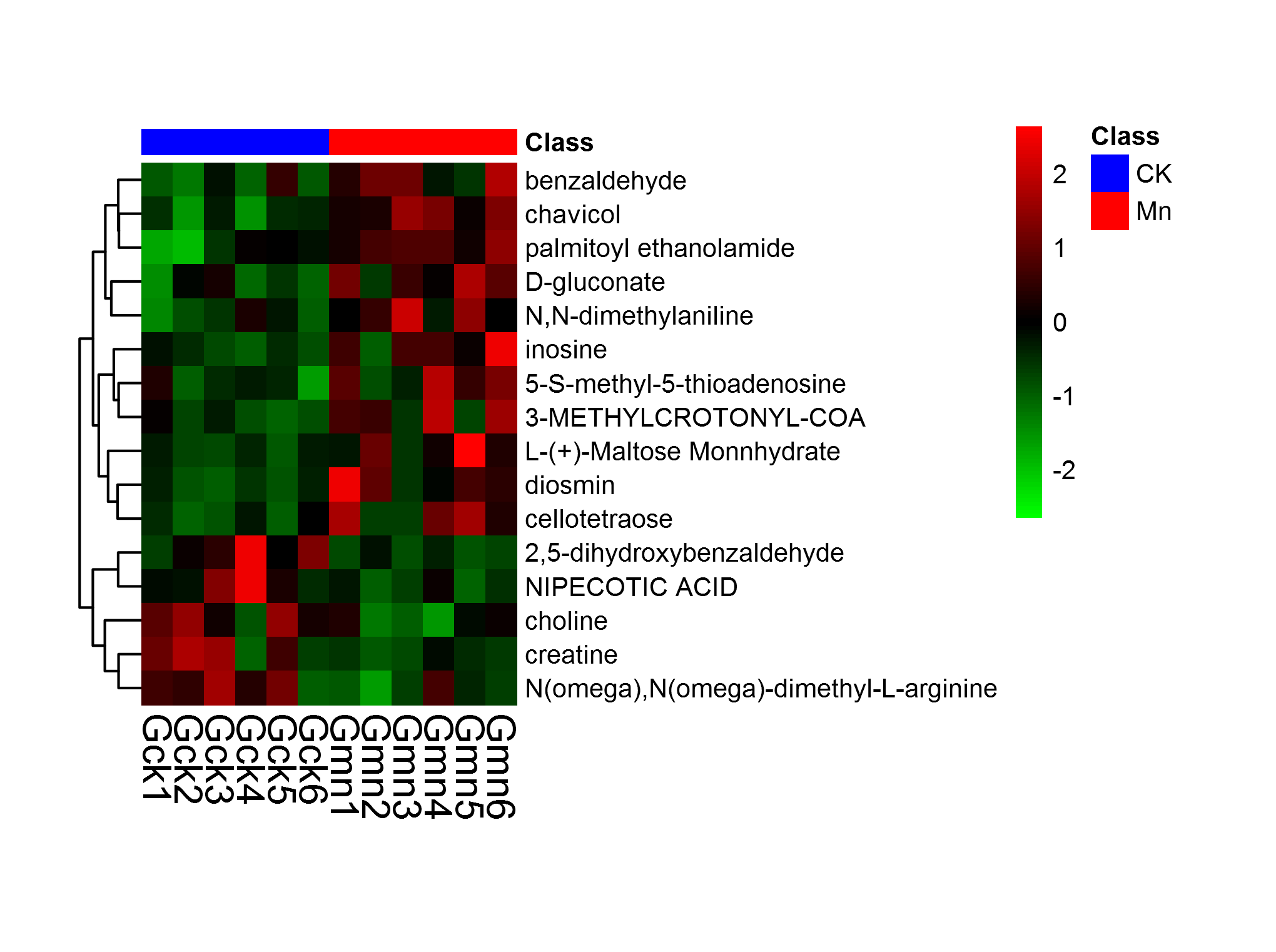

Supplement: Supplemental Information 3 — The raw data were for the LC-MS analysis including PLS-DA analysis in both positive and negative ionization mode, significantly differential metabolites of Ganoderma lucidum between treatments, mutual promotion or inhibition relationships between differential metabolites, etc. [file peerj-07-6846-s003.zip › raw data/CK vs Mn/cluster/heatmap_dem.png]

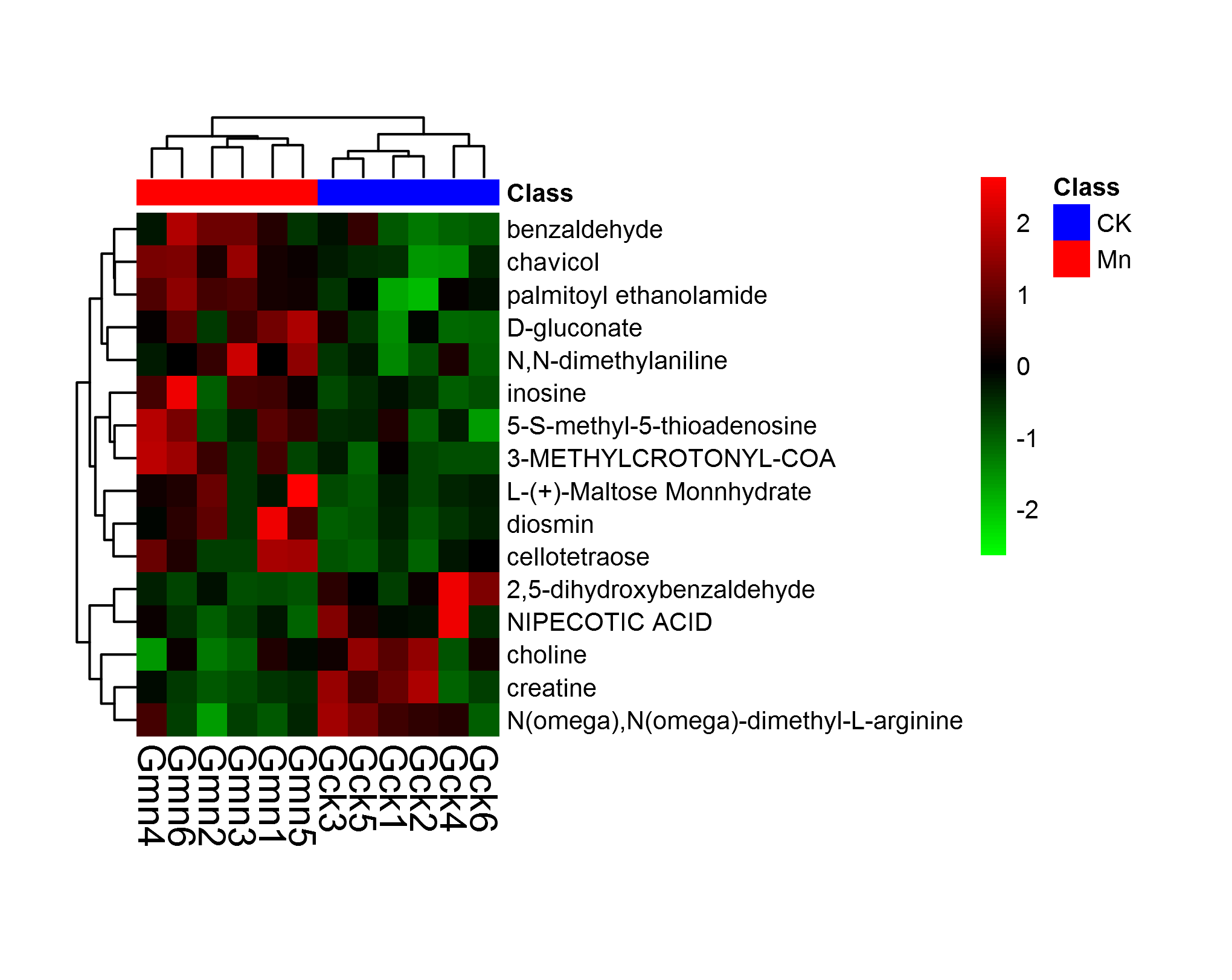

Supplement: Supplemental Information 3 — The raw data were for the LC-MS analysis including PLS-DA analysis in both positive and negative ionization mode, significantly differential metabolites of Ganoderma lucidum between treatments, mutual promotion or inhibition relationships between differential metabolites, etc. [file peerj-07-6846-s003.zip › raw data/CK vs Mn/cluster/heatmap_dem_cluster.png]

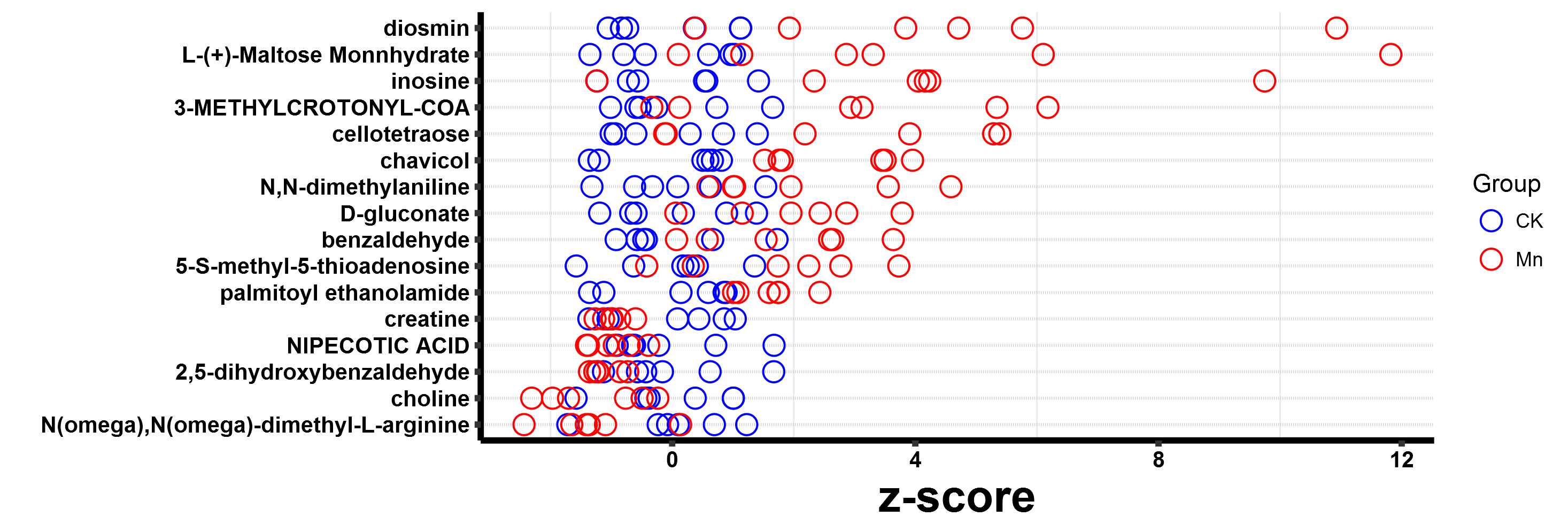

Supplement: Supplemental Information 3 — The raw data were for the LC-MS analysis including PLS-DA analysis in both positive and negative ionization mode, significantly differential metabolites of Ganoderma lucidum between treatments, mutual promotion or inhibition relationships between differential metabolites, etc. [file peerj-07-6846-s003.zip › raw data/CK vs Mn/DAM/z-score_dem.png]

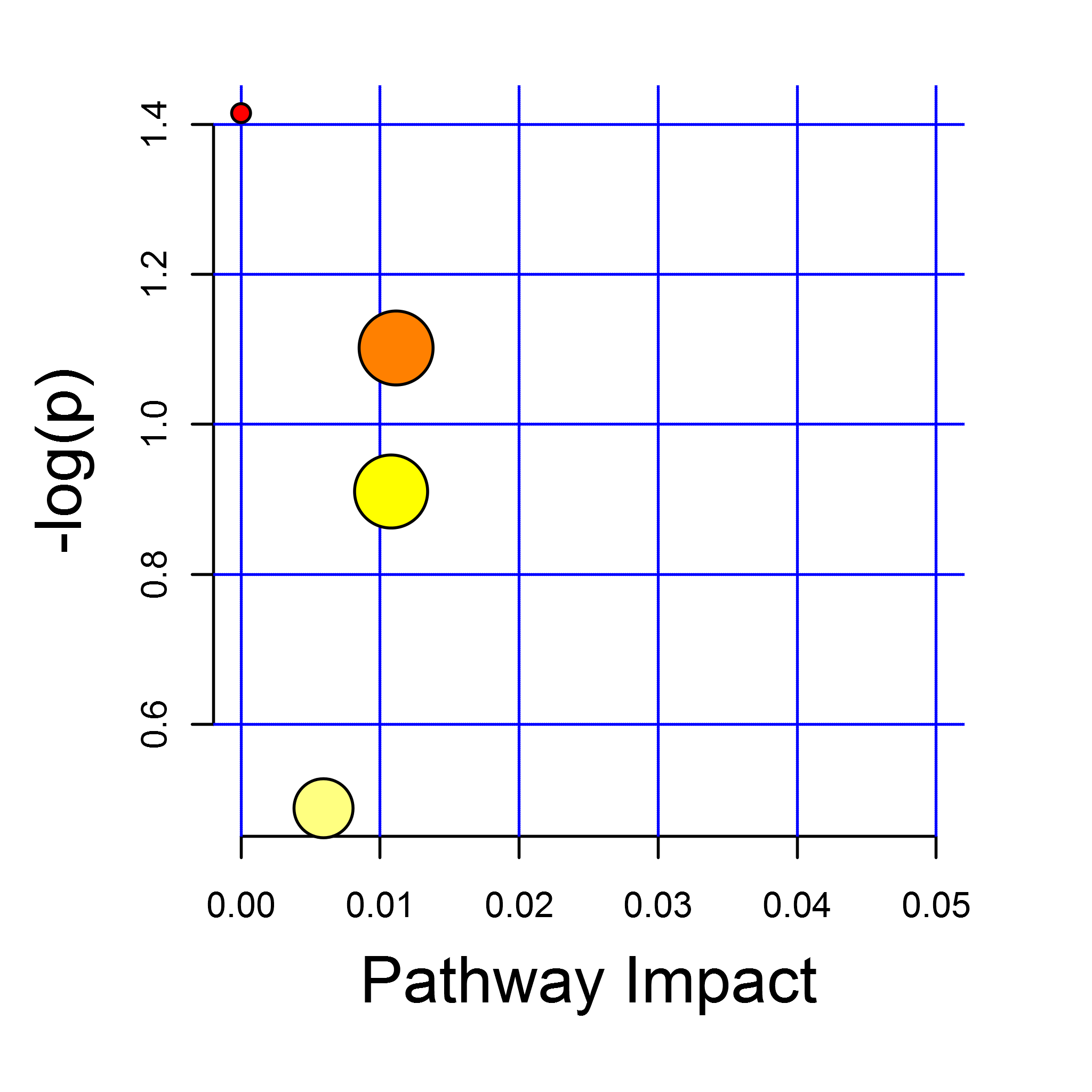

Supplement: Supplemental Information 3 — The raw data were for the LC-MS analysis including PLS-DA analysis in both positive and negative ionization mode, significantly differential metabolites of Ganoderma lucidum between treatments, mutual promotion or inhibition relationships between differential metabolites, etc. [file peerj-07-6846-s003.zip › raw data/CK vs Mn/pathway/KEGG_enrich.png]

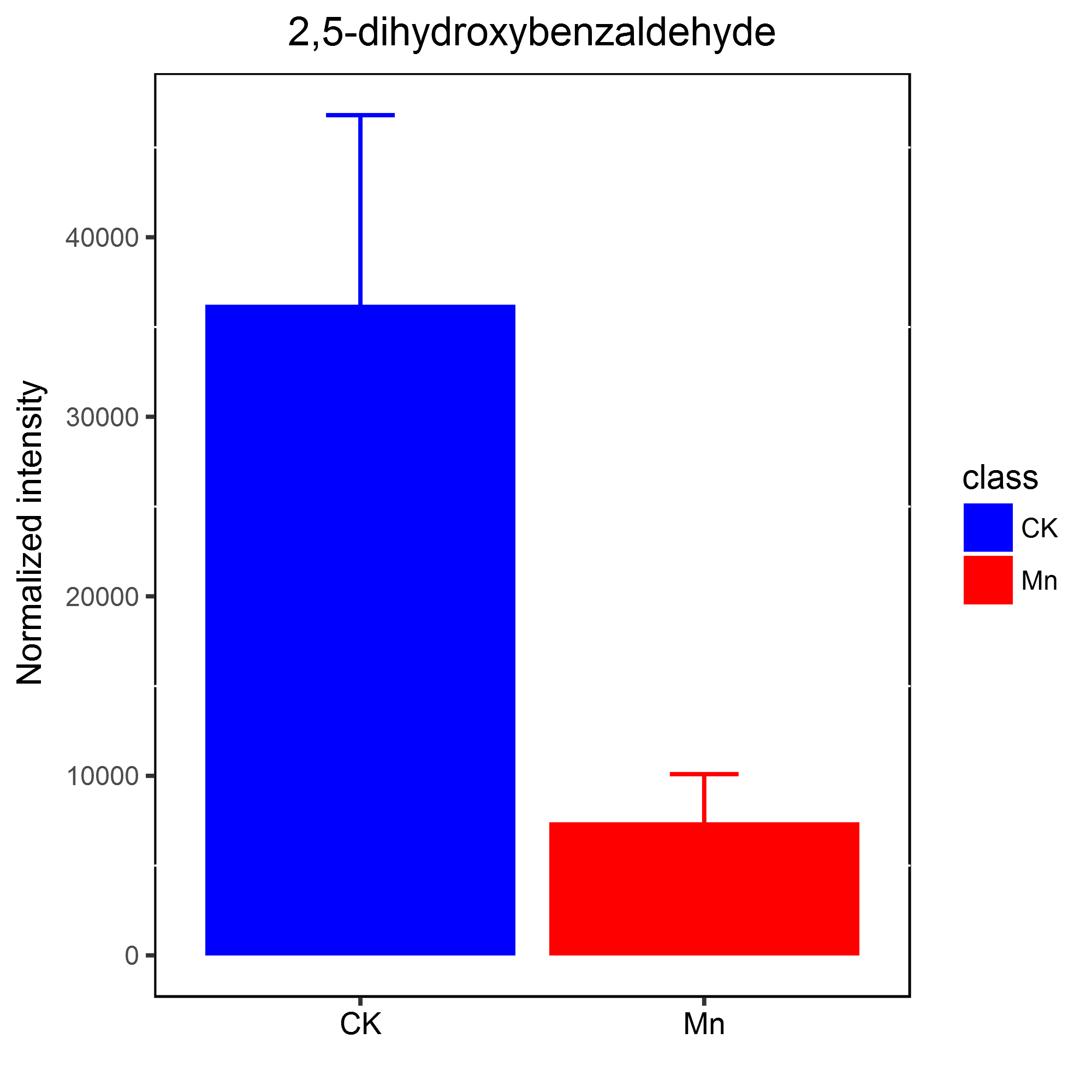

Supplement: Supplemental Information 3 — The raw data were for the LC-MS analysis including PLS-DA analysis in both positive and negative ionization mode, significantly differential metabolites of Ganoderma lucidum between treatments, mutual promotion or inhibition relationships between differential metabolites, etc. [file peerj-07-6846-s003.zip › raw data/CK vs Mn/visual/bar/2,5-dihydroxybenzaldehyde.png]

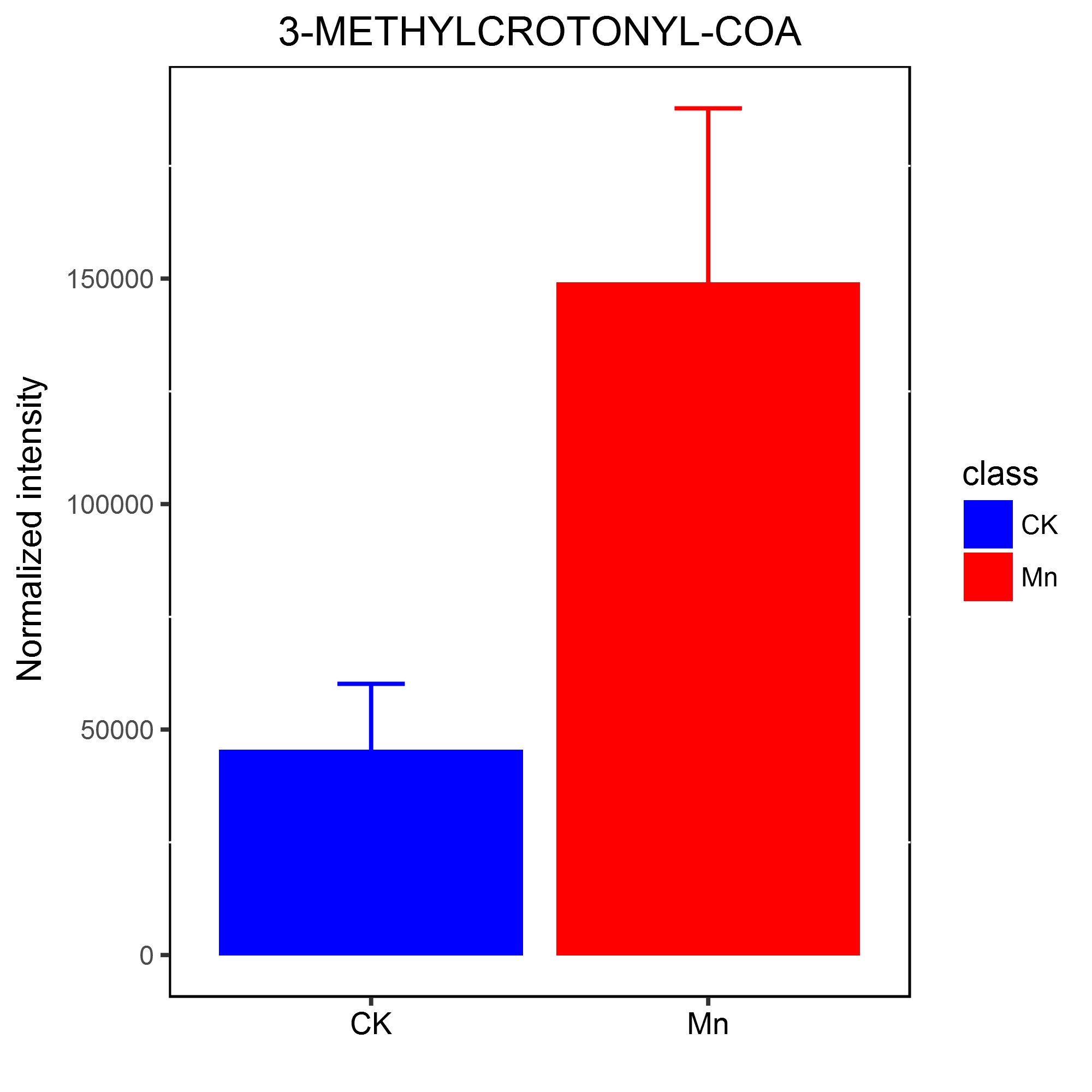

Supplement: Supplemental Information 3 — The raw data were for the LC-MS analysis including PLS-DA analysis in both positive and negative ionization mode, significantly differential metabolites of Ganoderma lucidum between treatments, mutual promotion or inhibition relationships between differential metabolites, etc. [file peerj-07-6846-s003.zip › raw data/CK vs Mn/visual/bar/3-METHYLCROTONYL-COA.png]

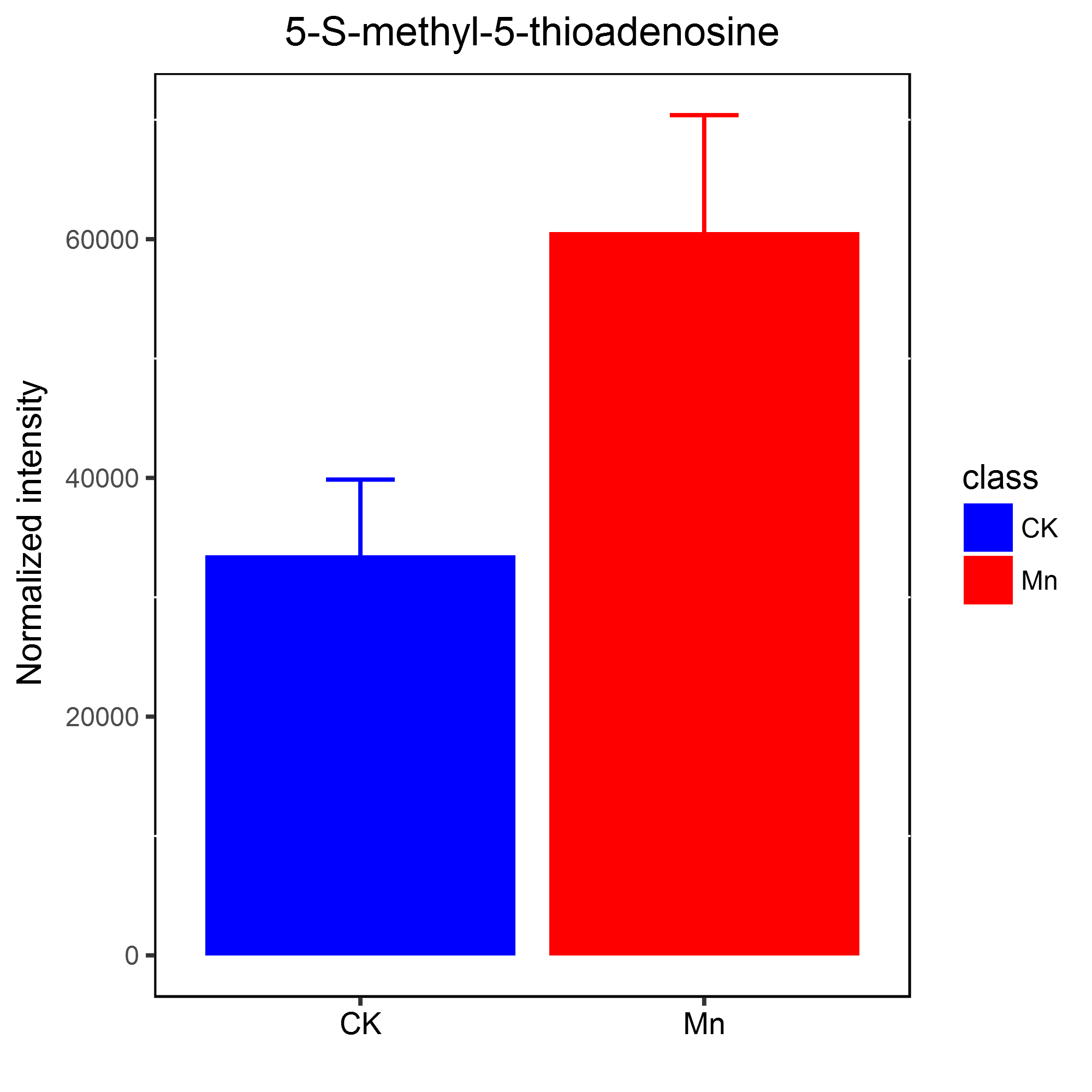

Supplement: Supplemental Information 3 — The raw data were for the LC-MS analysis including PLS-DA analysis in both positive and negative ionization mode, significantly differential metabolites of Ganoderma lucidum between treatments, mutual promotion or inhibition relationships between differential metabolites, etc. [file peerj-07-6846-s003.zip › raw data/CK vs Mn/visual/bar/5-S-methyl-5-thioadenosine.png]

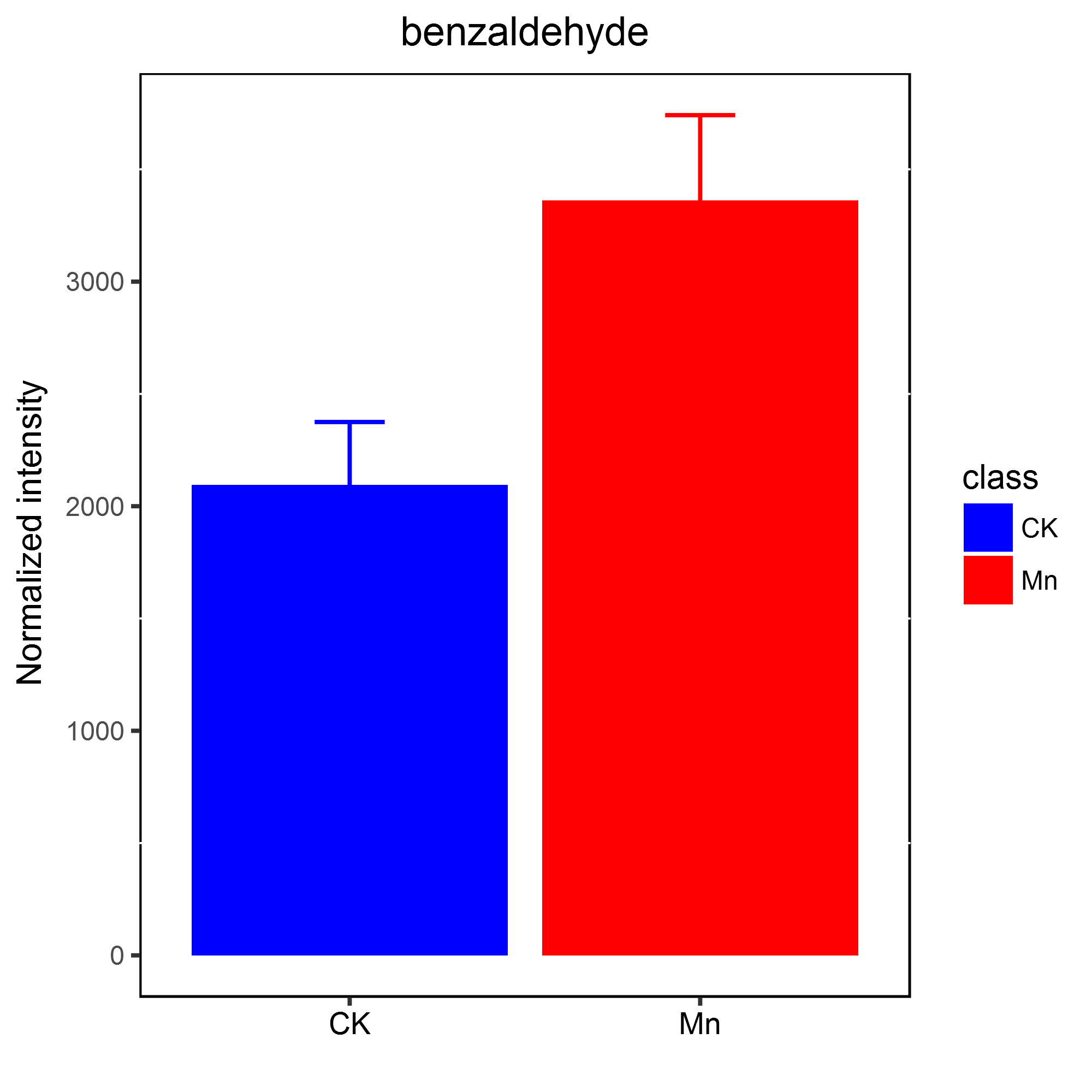

Supplement: Supplemental Information 3 — The raw data were for the LC-MS analysis including PLS-DA analysis in both positive and negative ionization mode, significantly differential metabolites of Ganoderma lucidum between treatments, mutual promotion or inhibition relationships between differential metabolites, etc. [file peerj-07-6846-s003.zip › raw data/CK vs Mn/visual/bar/benzaldehyde.png]

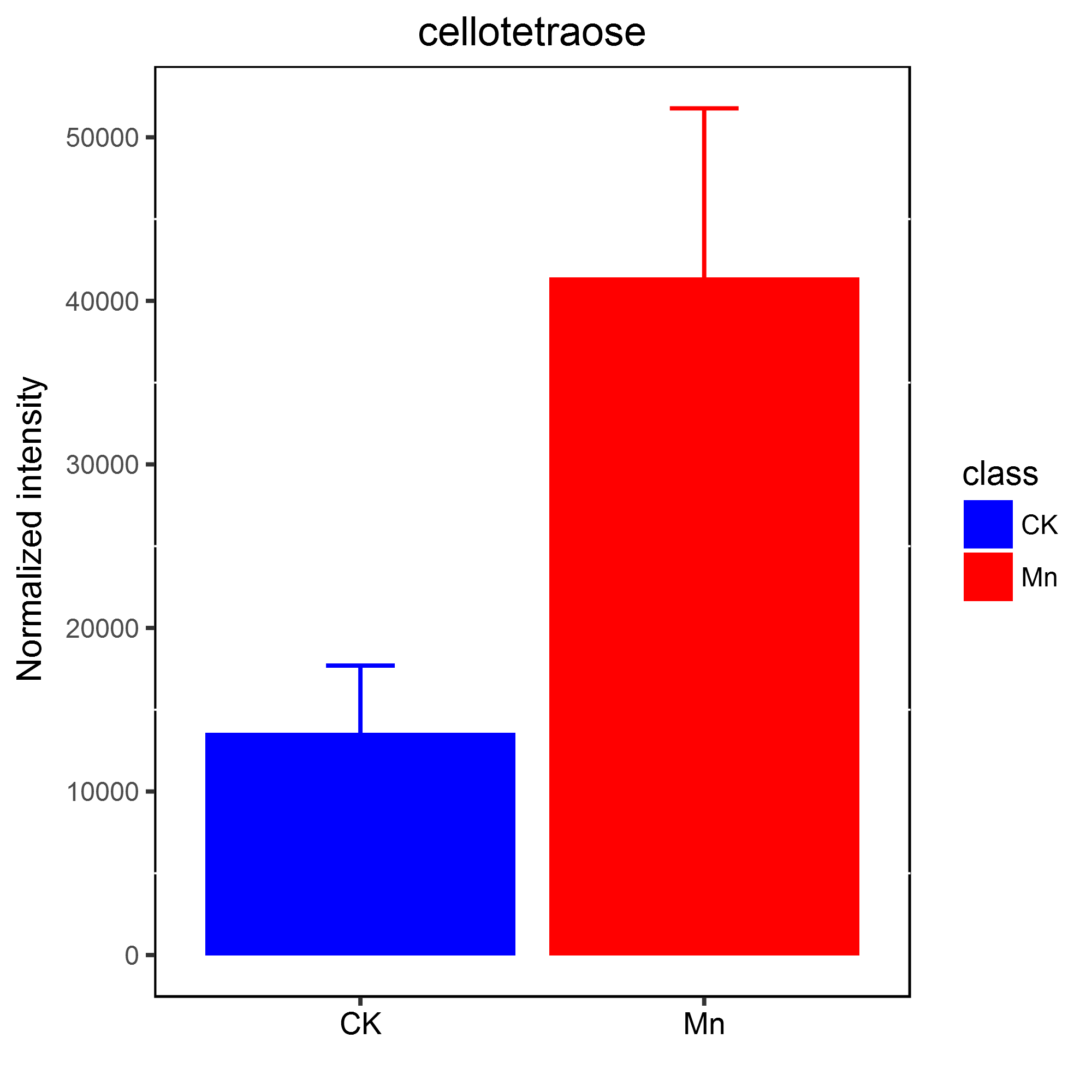

Supplement: Supplemental Information 3 — The raw data were for the LC-MS analysis including PLS-DA analysis in both positive and negative ionization mode, significantly differential metabolites of Ganoderma lucidum between treatments, mutual promotion or inhibition relationships between differential metabolites, etc. [file peerj-07-6846-s003.zip › raw data/CK vs Mn/visual/bar/cellotetraose.png]

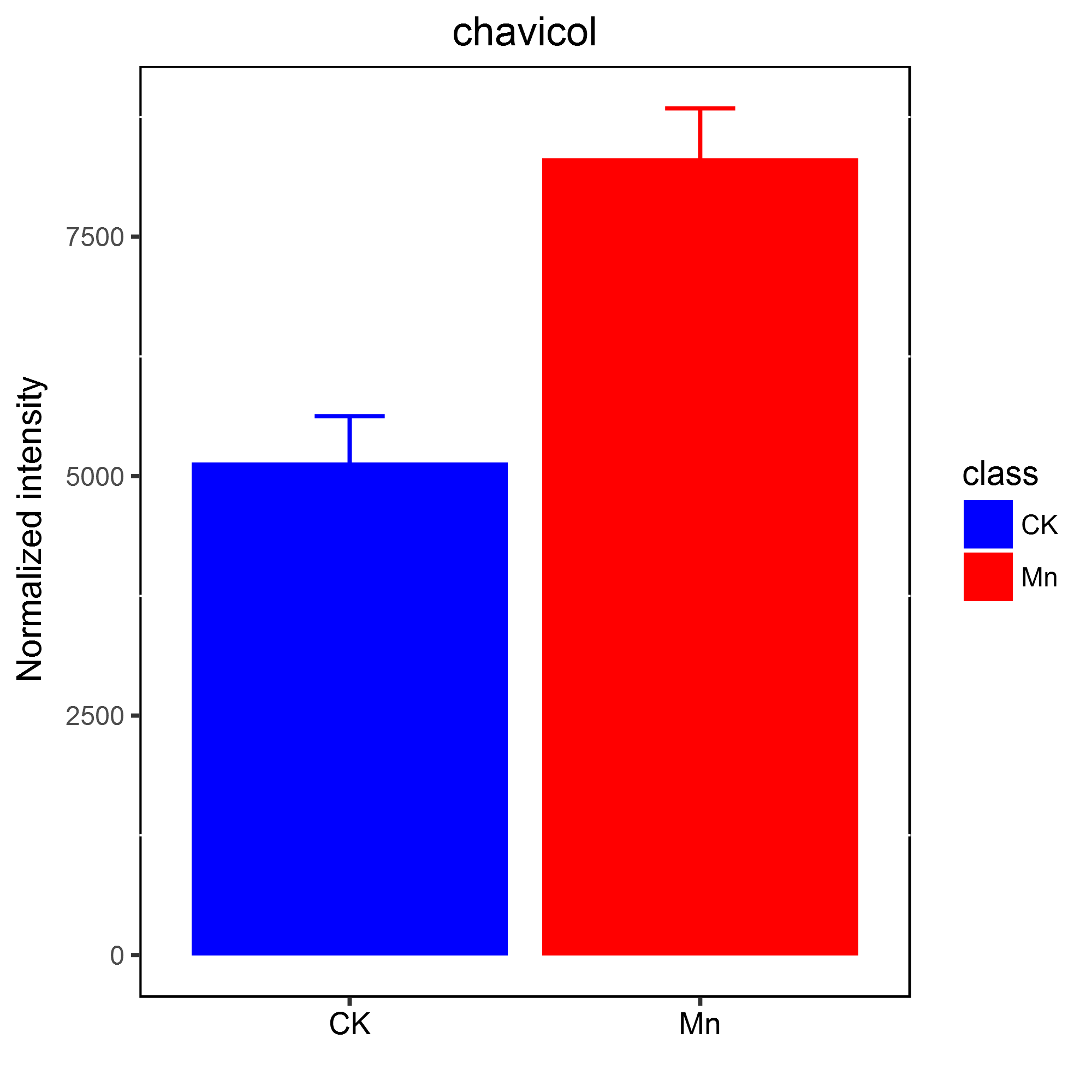

Supplement: Supplemental Information 3 — The raw data were for the LC-MS analysis including PLS-DA analysis in both positive and negative ionization mode, significantly differential metabolites of Ganoderma lucidum between treatments, mutual promotion or inhibition relationships between differential metabolites, etc. [file peerj-07-6846-s003.zip › raw data/CK vs Mn/visual/bar/chavicol.png]

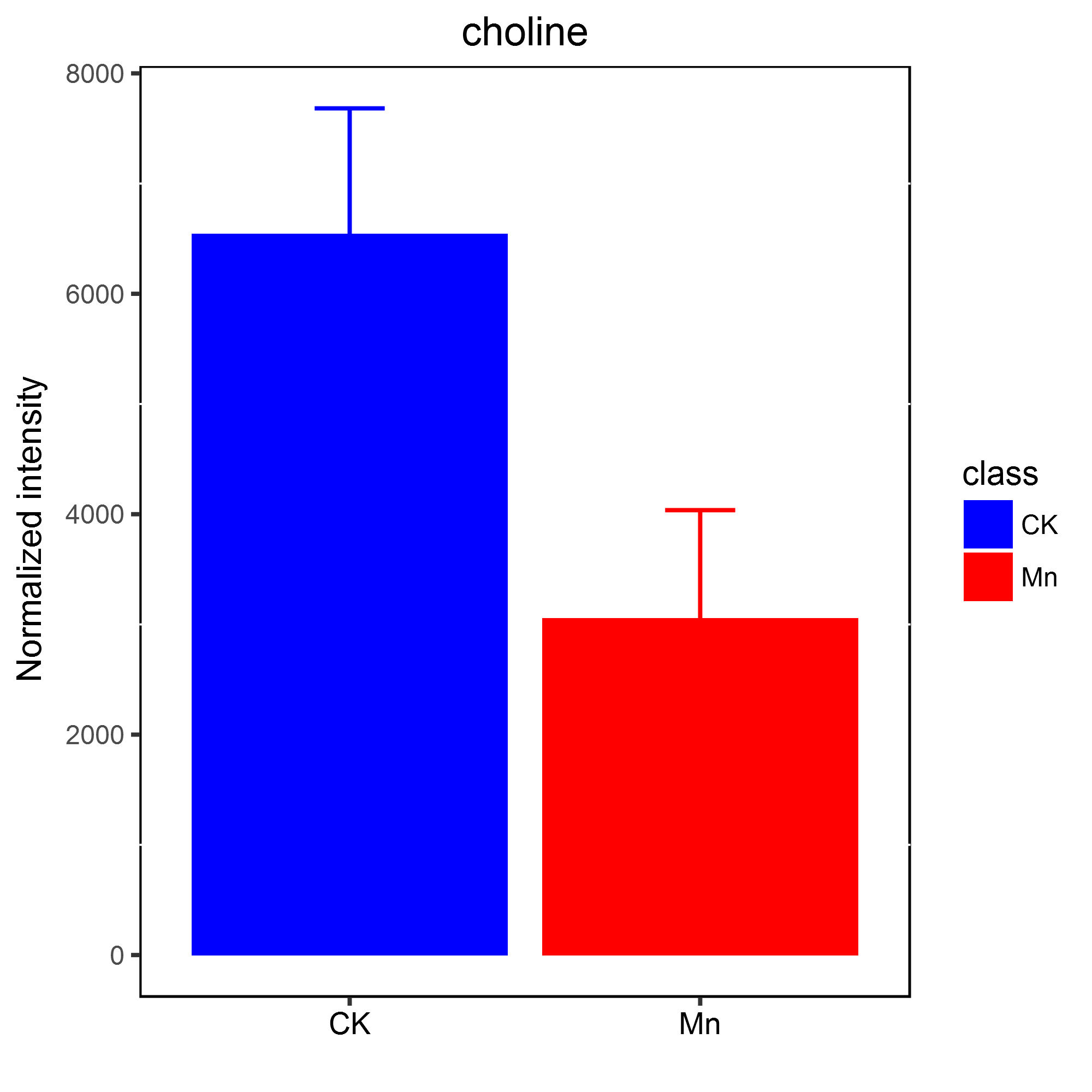

Supplement: Supplemental Information 3 — The raw data were for the LC-MS analysis including PLS-DA analysis in both positive and negative ionization mode, significantly differential metabolites of Ganoderma lucidum between treatments, mutual promotion or inhibition relationships between differential metabolites, etc. [file peerj-07-6846-s003.zip › raw data/CK vs Mn/visual/bar/choline.png]

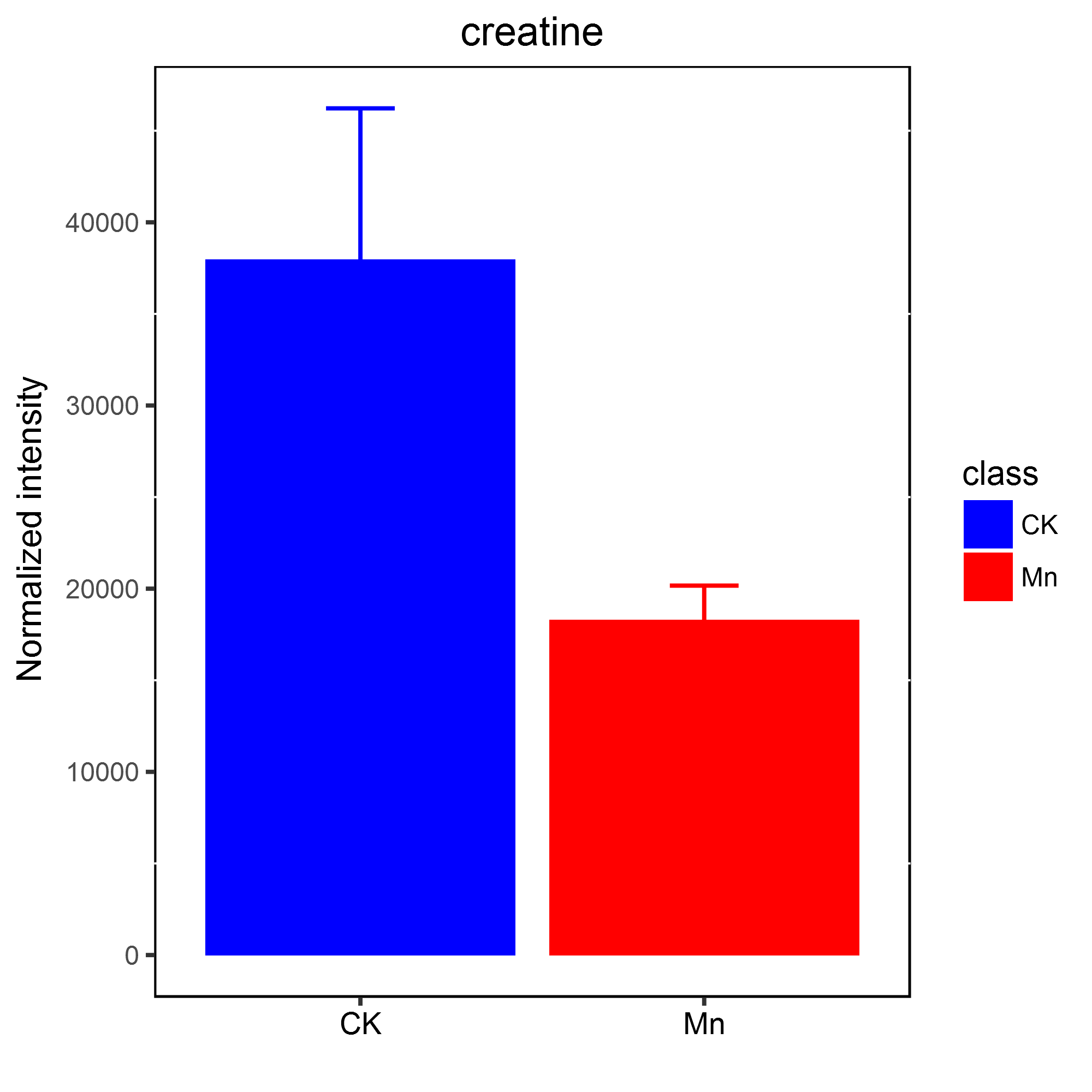

Supplement: Supplemental Information 3 — The raw data were for the LC-MS analysis including PLS-DA analysis in both positive and negative ionization mode, significantly differential metabolites of Ganoderma lucidum between treatments, mutual promotion or inhibition relationships between differential metabolites, etc. [file peerj-07-6846-s003.zip › raw data/CK vs Mn/visual/bar/creatine.png]

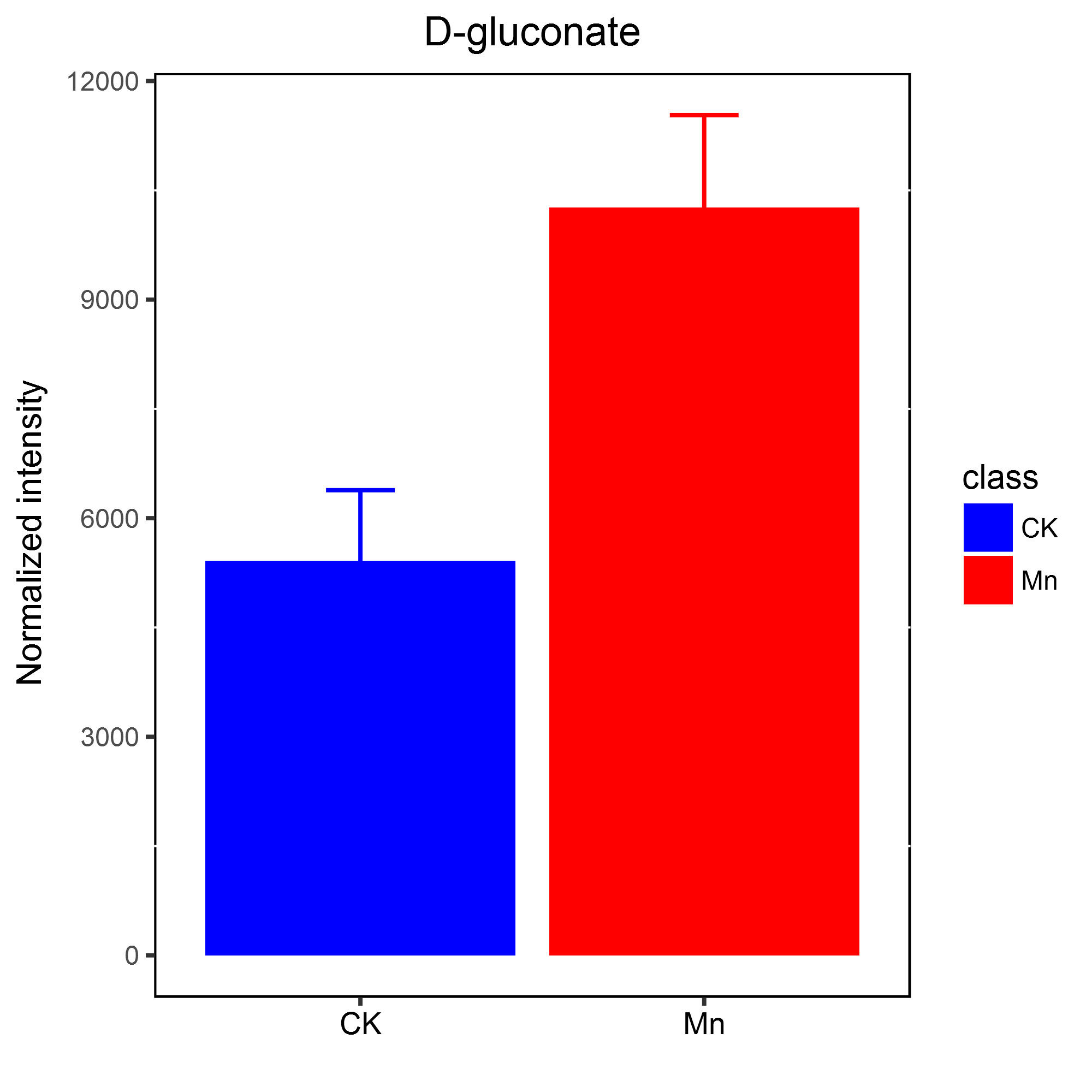

Supplement: Supplemental Information 3 — The raw data were for the LC-MS analysis including PLS-DA analysis in both positive and negative ionization mode, significantly differential metabolites of Ganoderma lucidum between treatments, mutual promotion or inhibition relationships between differential metabolites, etc. [file peerj-07-6846-s003.zip › raw data/CK vs Mn/visual/bar/D-gluconate.png]

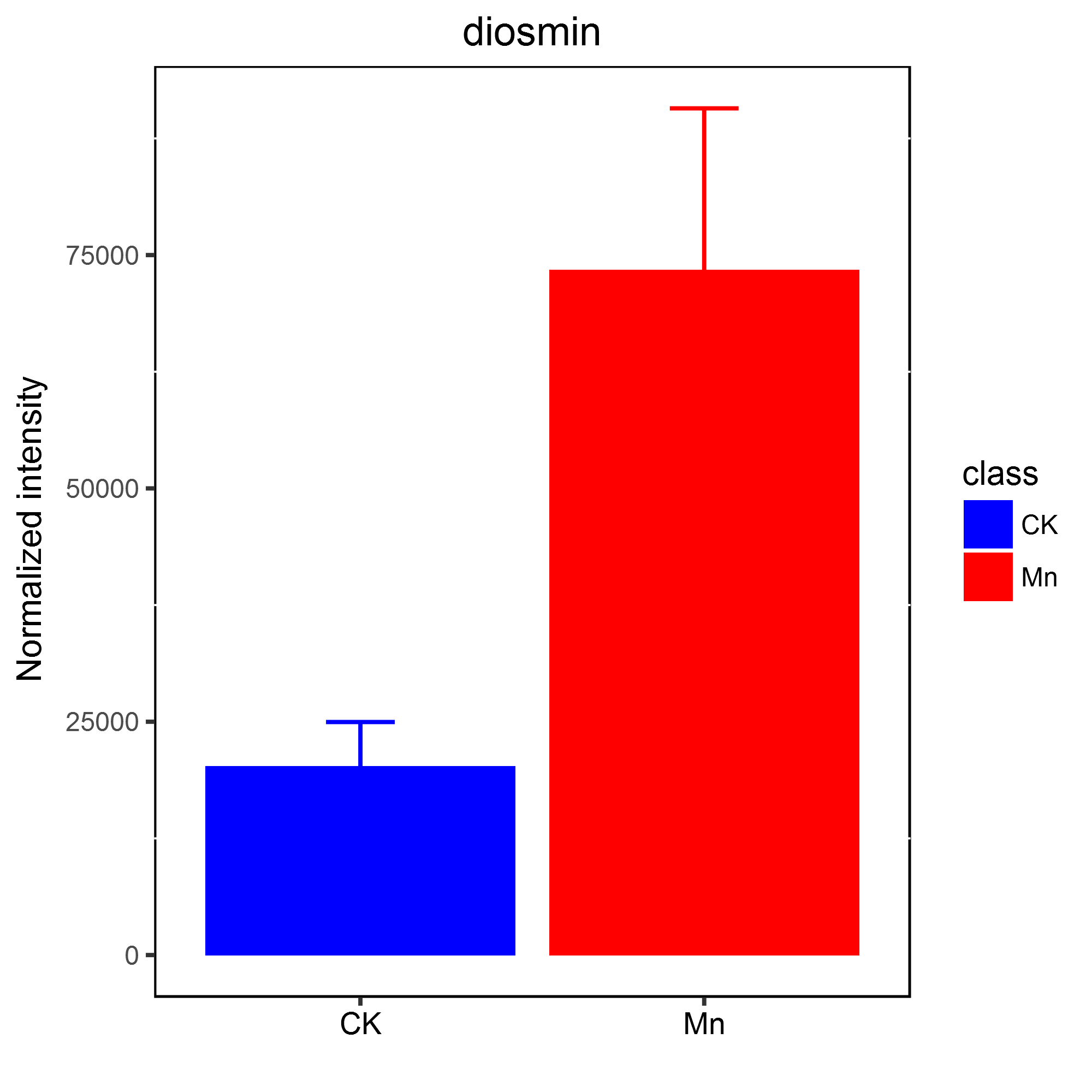

Supplement: Supplemental Information 3 — The raw data were for the LC-MS analysis including PLS-DA analysis in both positive and negative ionization mode, significantly differential metabolites of Ganoderma lucidum between treatments, mutual promotion or inhibition relationships between differential metabolites, etc. [file peerj-07-6846-s003.zip › raw data/CK vs Mn/visual/bar/diosmin.png]

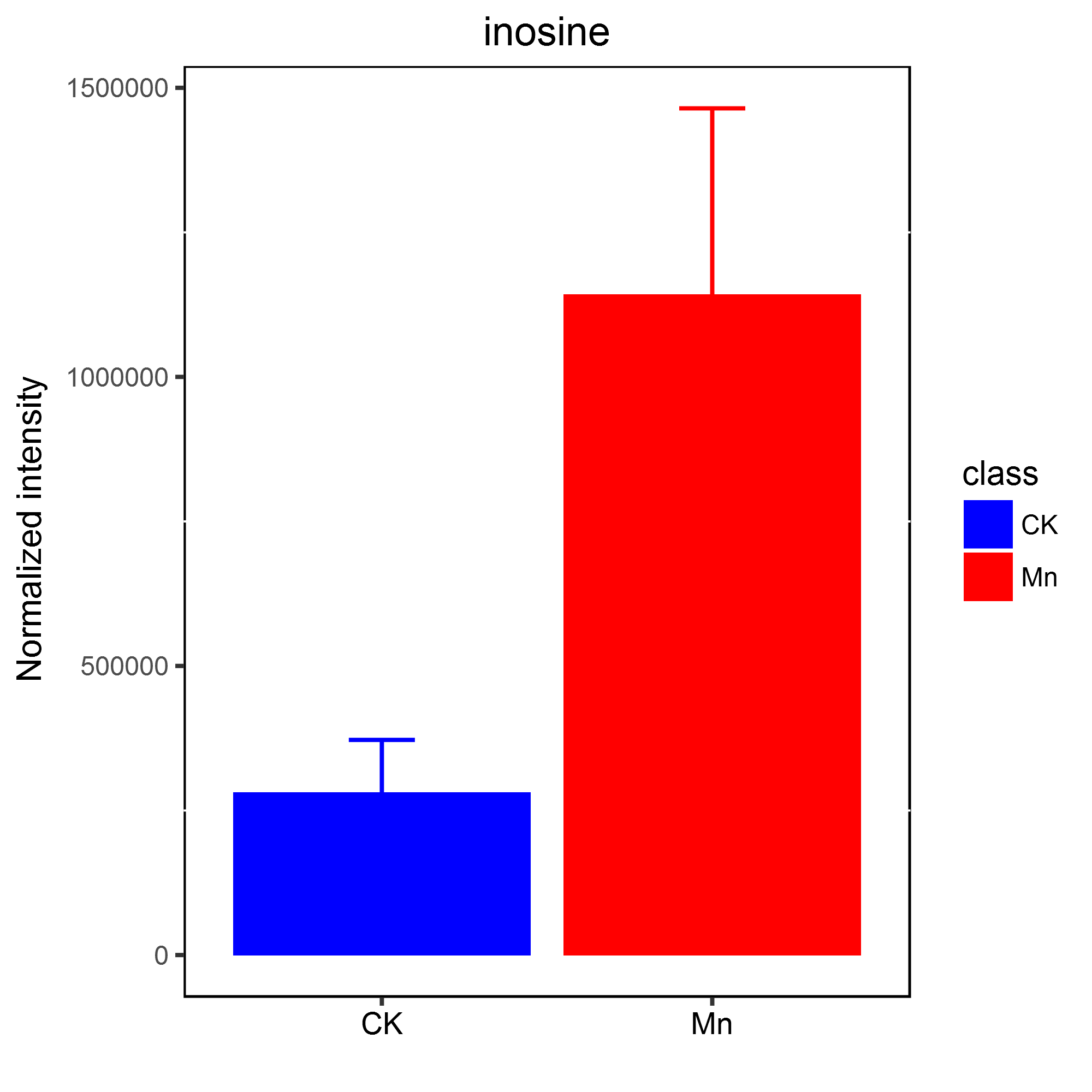

Supplement: Supplemental Information 3 — The raw data were for the LC-MS analysis including PLS-DA analysis in both positive and negative ionization mode, significantly differential metabolites of Ganoderma lucidum between treatments, mutual promotion or inhibition relationships between differential metabolites, etc. [file peerj-07-6846-s003.zip › raw data/CK vs Mn/visual/bar/inosine.png]

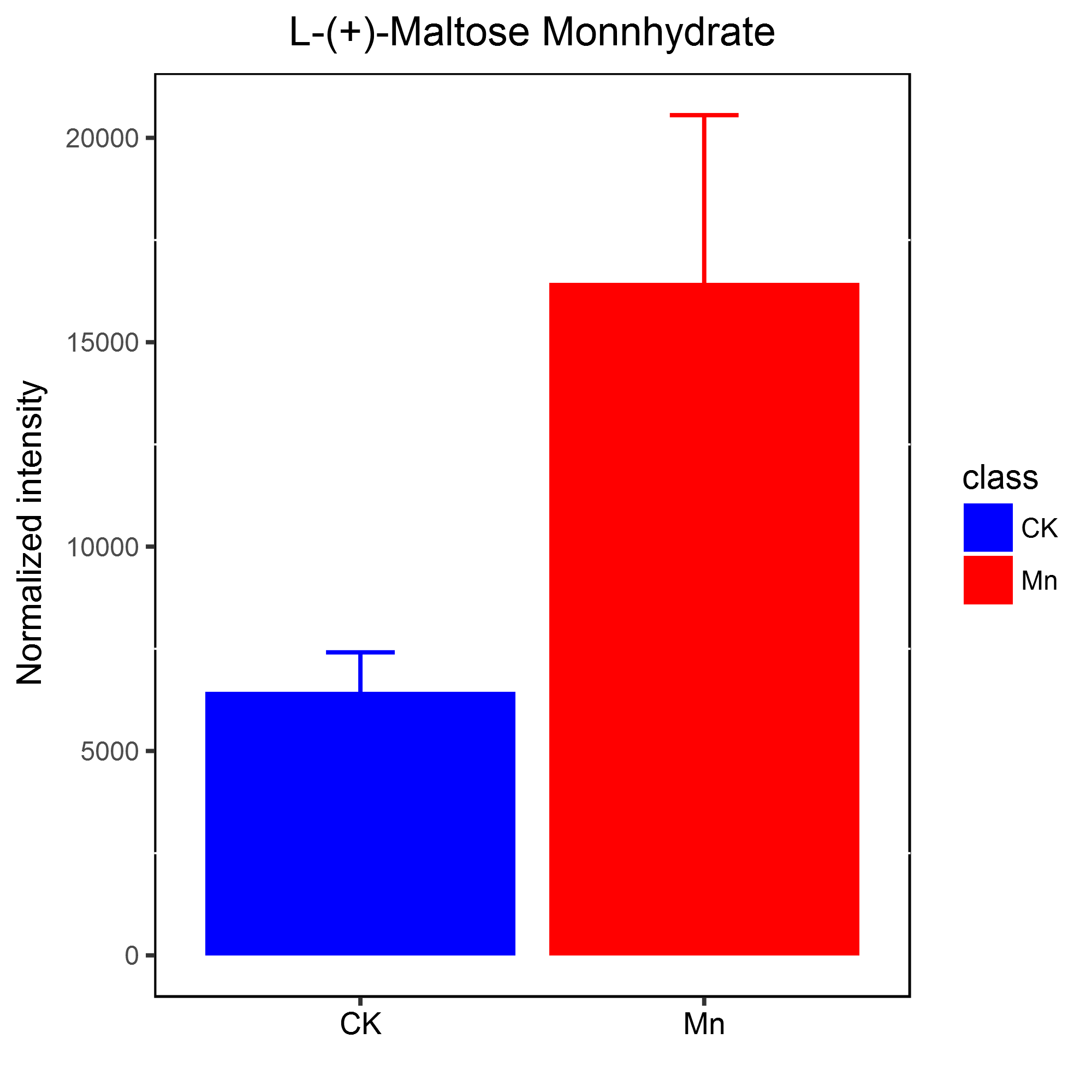

Supplement: Supplemental Information 3 — The raw data were for the LC-MS analysis including PLS-DA analysis in both positive and negative ionization mode, significantly differential metabolites of Ganoderma lucidum between treatments, mutual promotion or inhibition relationships between differential metabolites, etc. [file peerj-07-6846-s003.zip › raw data/CK vs Mn/visual/bar/L-(+)-Maltose Monnhydrate.png]

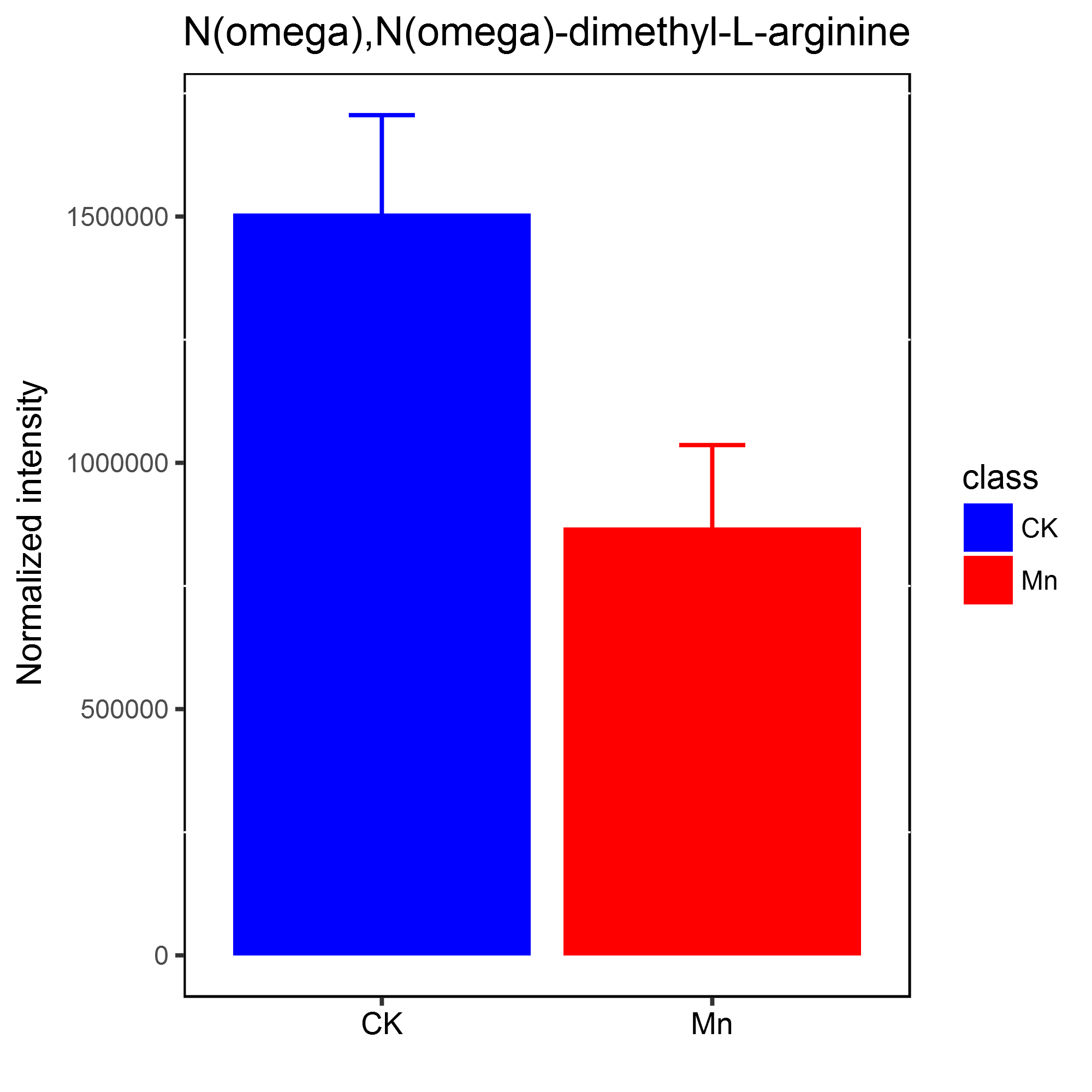

Supplement: Supplemental Information 3 — The raw data were for the LC-MS analysis including PLS-DA analysis in both positive and negative ionization mode, significantly differential metabolites of Ganoderma lucidum between treatments, mutual promotion or inhibition relationships between differential metabolites, etc. [file peerj-07-6846-s003.zip › raw data/CK vs Mn/visual/bar/N(omega),N(omega)-dimethyl-L-arginine.png]

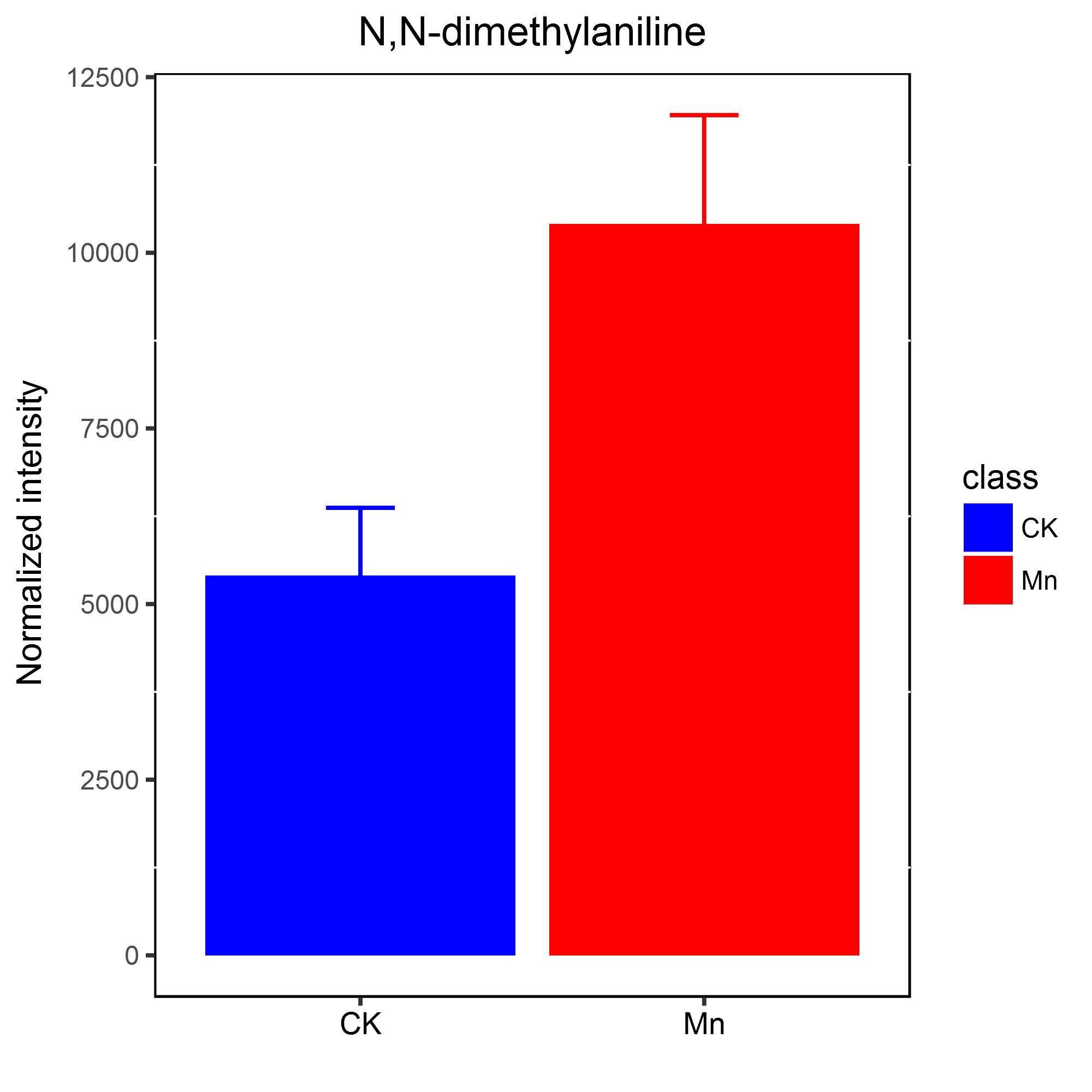

Supplement: Supplemental Information 3 — The raw data were for the LC-MS analysis including PLS-DA analysis in both positive and negative ionization mode, significantly differential metabolites of Ganoderma lucidum between treatments, mutual promotion or inhibition relationships between differential metabolites, etc. [file peerj-07-6846-s003.zip › raw data/CK vs Mn/visual/bar/N,N-dimethylaniline.png]

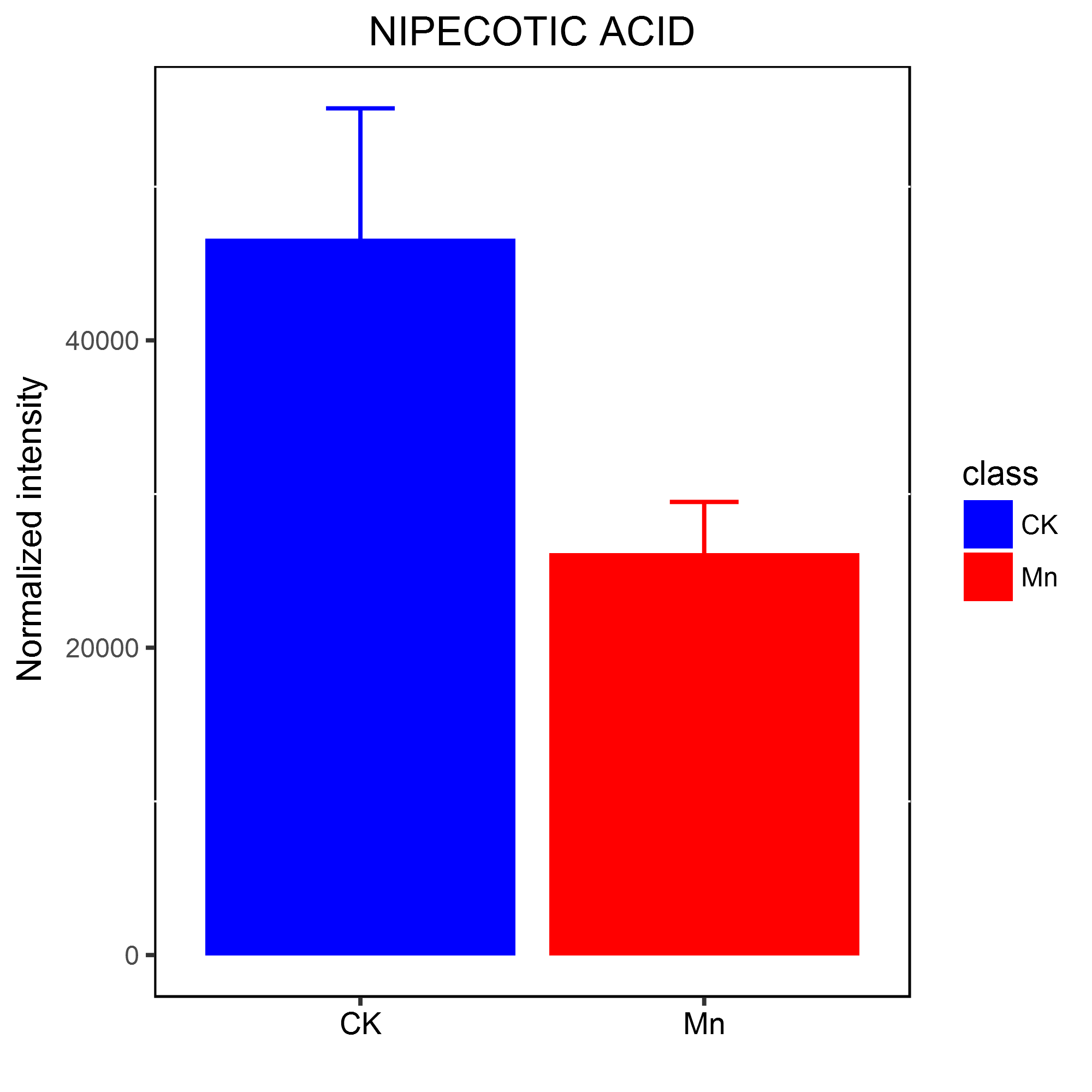

Supplement: Supplemental Information 3 — The raw data were for the LC-MS analysis including PLS-DA analysis in both positive and negative ionization mode, significantly differential metabolites of Ganoderma lucidum between treatments, mutual promotion or inhibition relationships between differential metabolites, etc. [file peerj-07-6846-s003.zip › raw data/CK vs Mn/visual/bar/NIPECOTIC ACID.png]

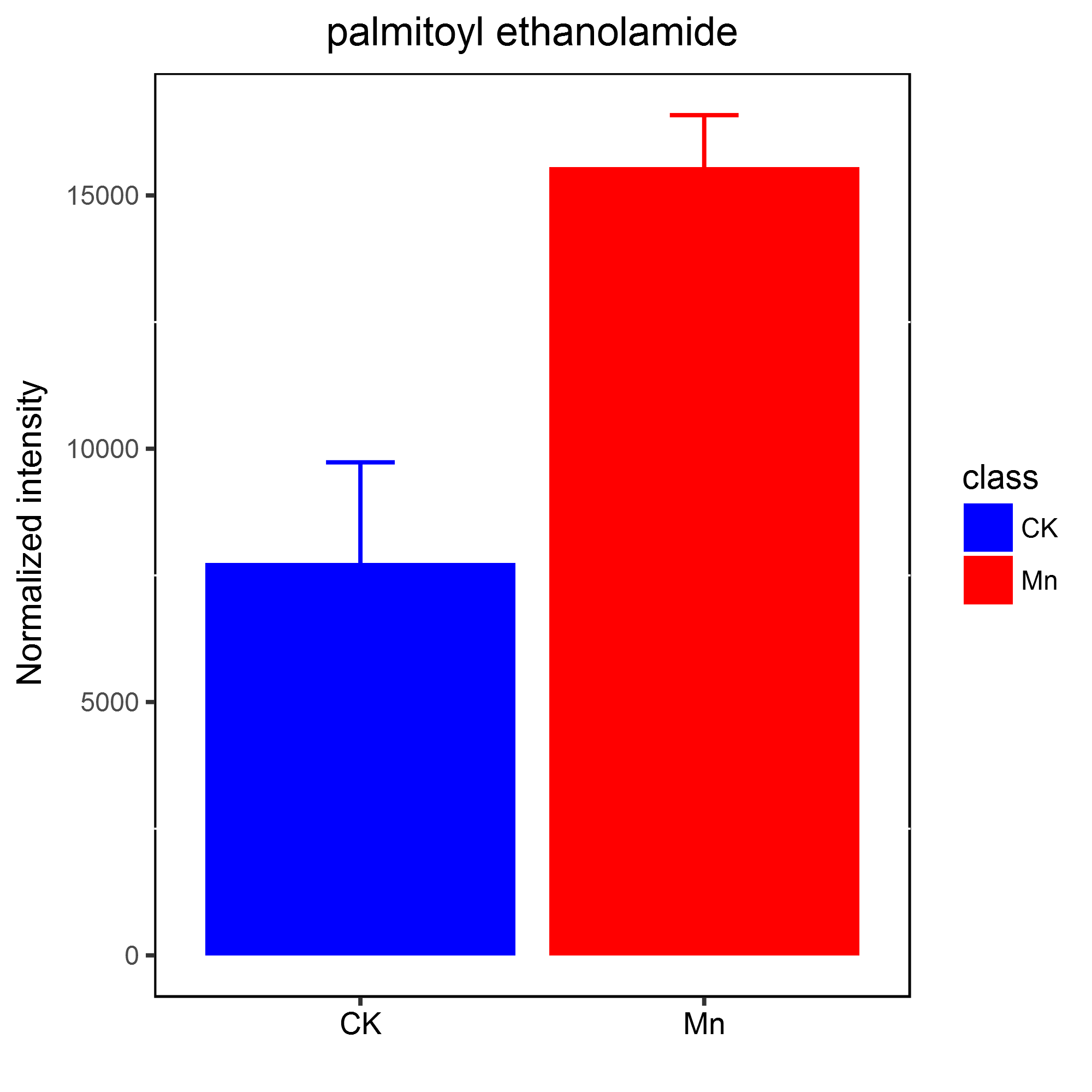

Supplement: Supplemental Information 3 — The raw data were for the LC-MS analysis including PLS-DA analysis in both positive and negative ionization mode, significantly differential metabolites of Ganoderma lucidum between treatments, mutual promotion or inhibition relationships between differential metabolites, etc. [file peerj-07-6846-s003.zip › raw data/CK vs Mn/visual/bar/palmitoyl ethanolamide.png]

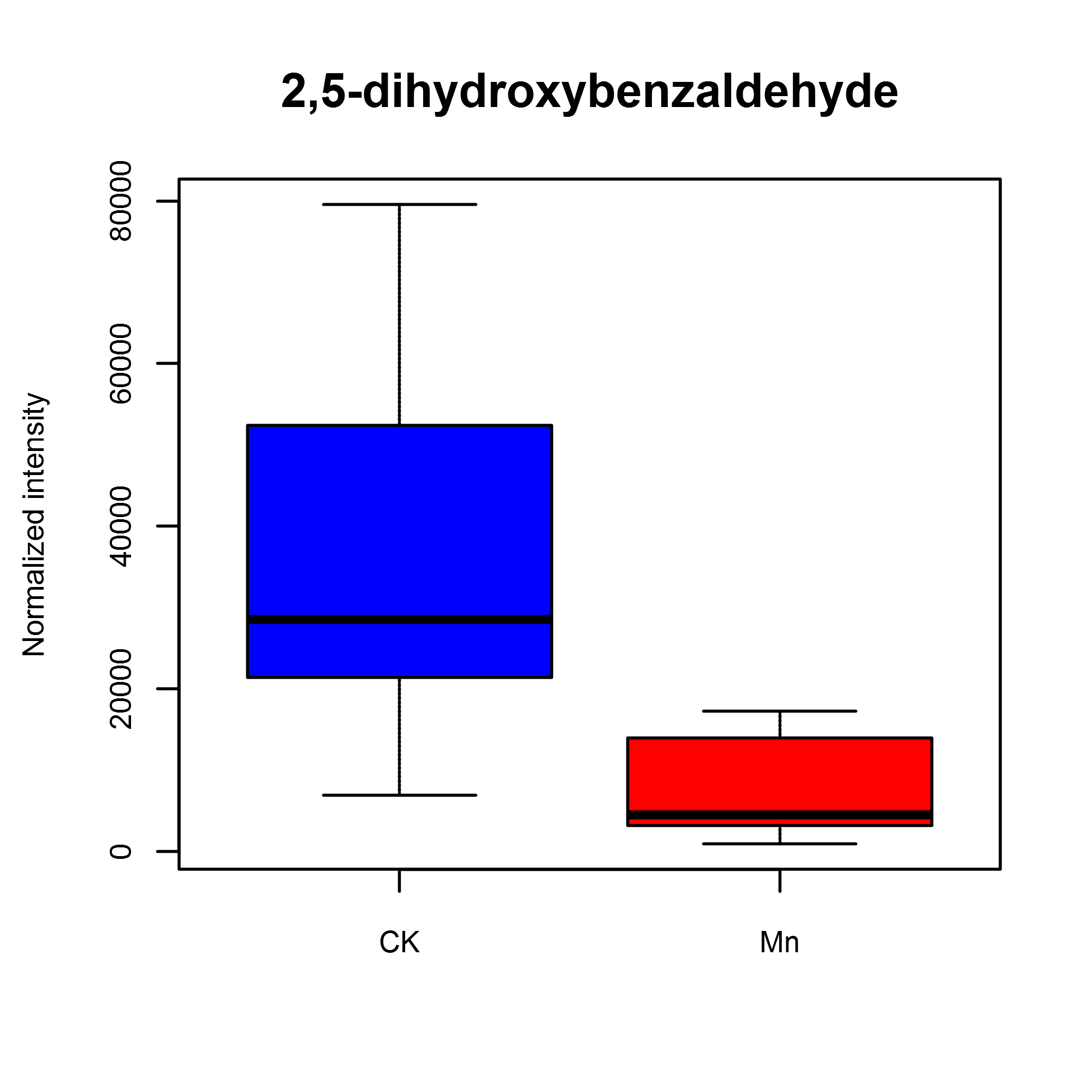

Supplement: Supplemental Information 3 — The raw data were for the LC-MS analysis including PLS-DA analysis in both positive and negative ionization mode, significantly differential metabolites of Ganoderma lucidum between treatments, mutual promotion or inhibition relationships between differential metabolites, etc. [file peerj-07-6846-s003.zip › raw data/CK vs Mn/visual/box/2,5-dihydroxybenzaldehyde.png]

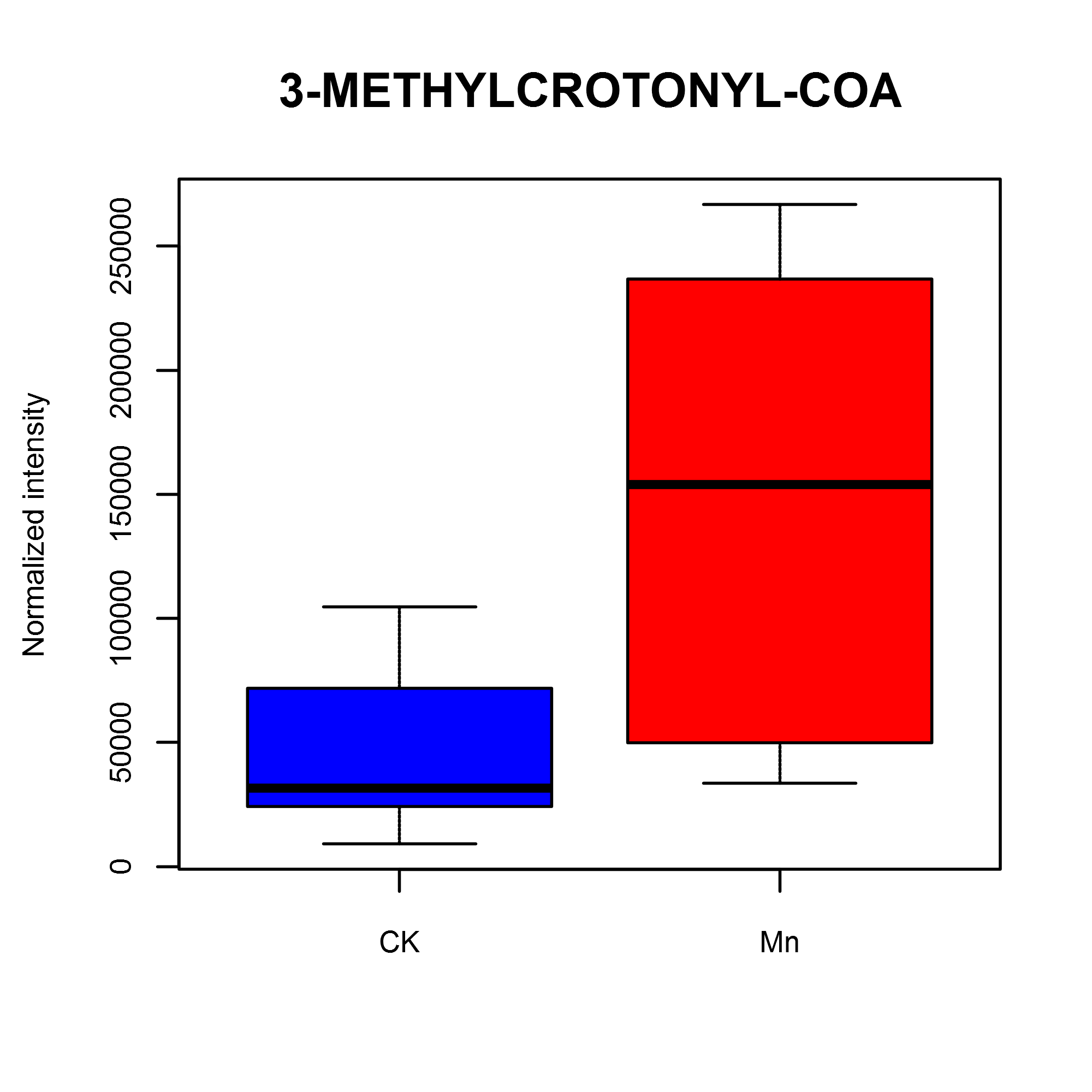

Supplement: Supplemental Information 3 — The raw data were for the LC-MS analysis including PLS-DA analysis in both positive and negative ionization mode, significantly differential metabolites of Ganoderma lucidum between treatments, mutual promotion or inhibition relationships between differential metabolites, etc. [file peerj-07-6846-s003.zip › raw data/CK vs Mn/visual/box/3-METHYLCROTONYL-COA.png]

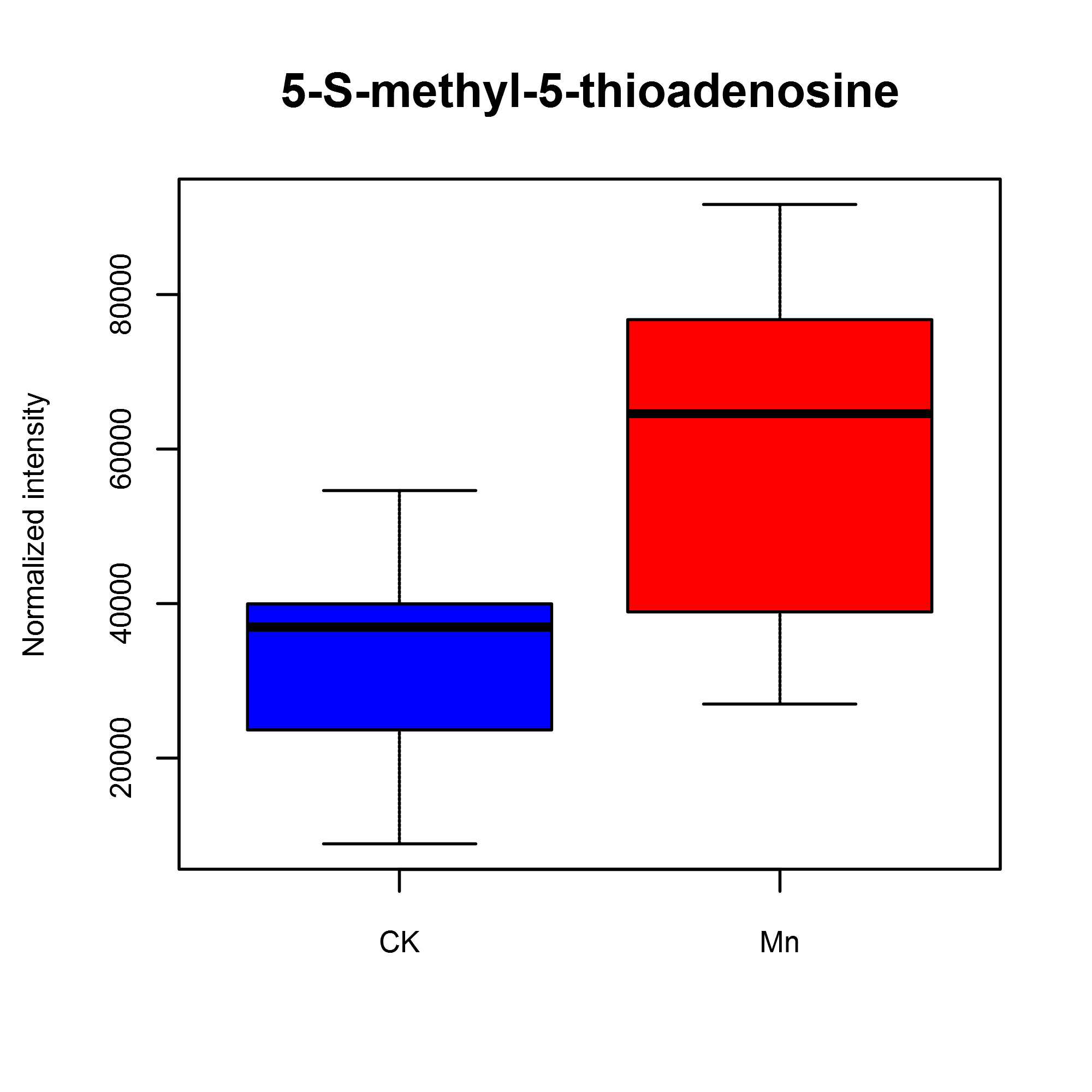

Supplement: Supplemental Information 3 — The raw data were for the LC-MS analysis including PLS-DA analysis in both positive and negative ionization mode, significantly differential metabolites of Ganoderma lucidum between treatments, mutual promotion or inhibition relationships between differential metabolites, etc. [file peerj-07-6846-s003.zip › raw data/CK vs Mn/visual/box/5-S-methyl-5-thioadenosine.png]

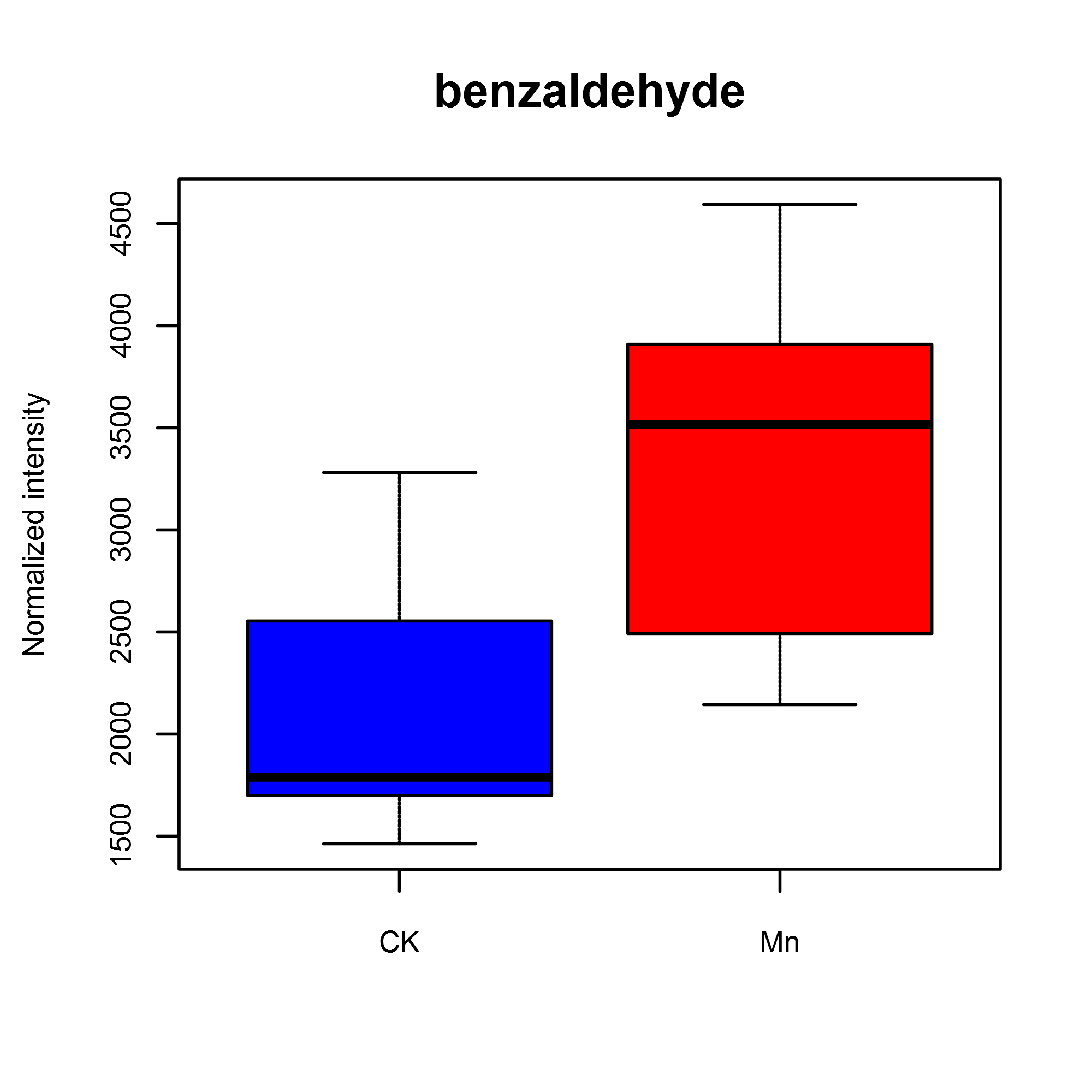

Supplement: Supplemental Information 3 — The raw data were for the LC-MS analysis including PLS-DA analysis in both positive and negative ionization mode, significantly differential metabolites of Ganoderma lucidum between treatments, mutual promotion or inhibition relationships between differential metabolites, etc. [file peerj-07-6846-s003.zip › raw data/CK vs Mn/visual/box/benzaldehyde.png]

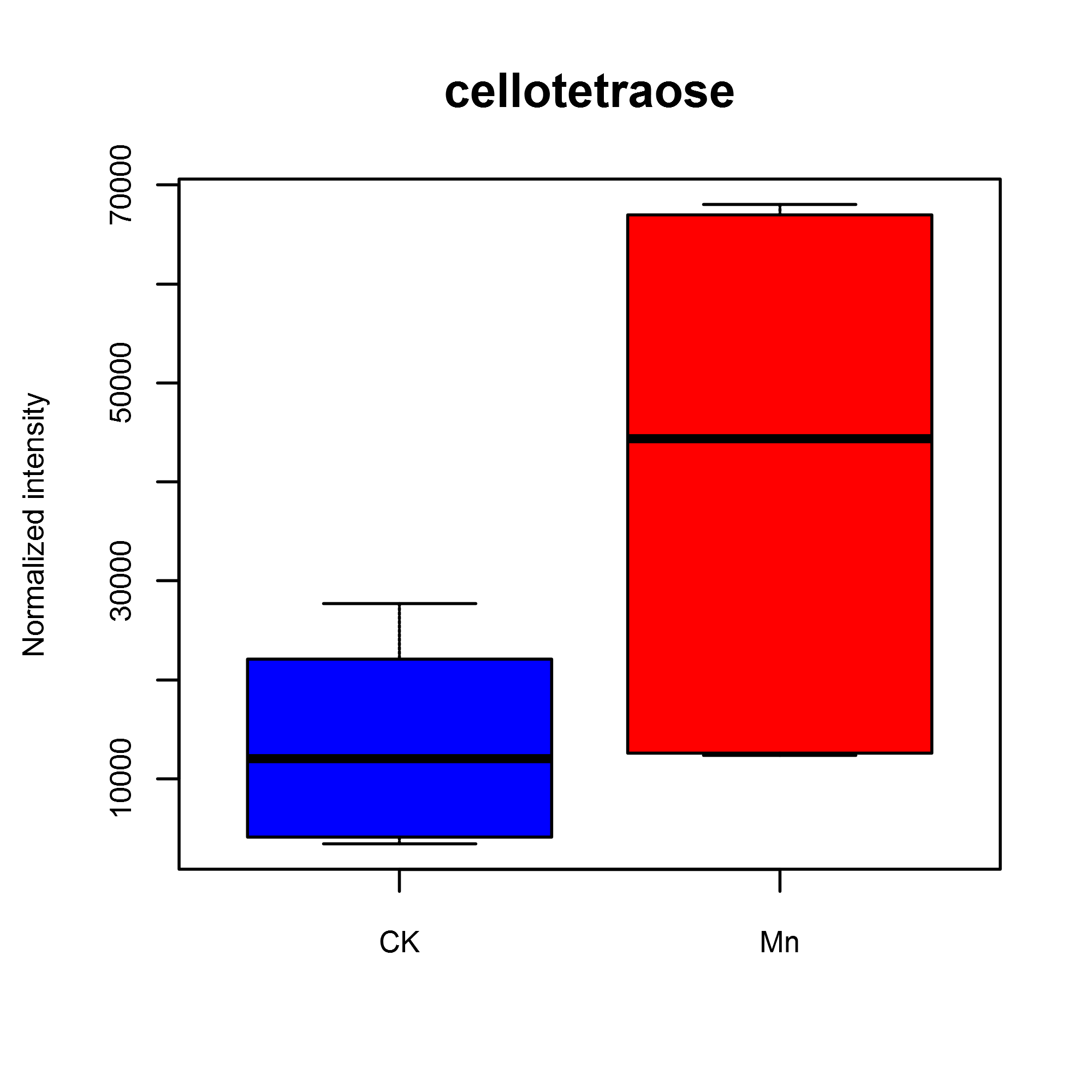

Supplement: Supplemental Information 3 — The raw data were for the LC-MS analysis including PLS-DA analysis in both positive and negative ionization mode, significantly differential metabolites of Ganoderma lucidum between treatments, mutual promotion or inhibition relationships between differential metabolites, etc. [file peerj-07-6846-s003.zip › raw data/CK vs Mn/visual/box/cellotetraose.png]

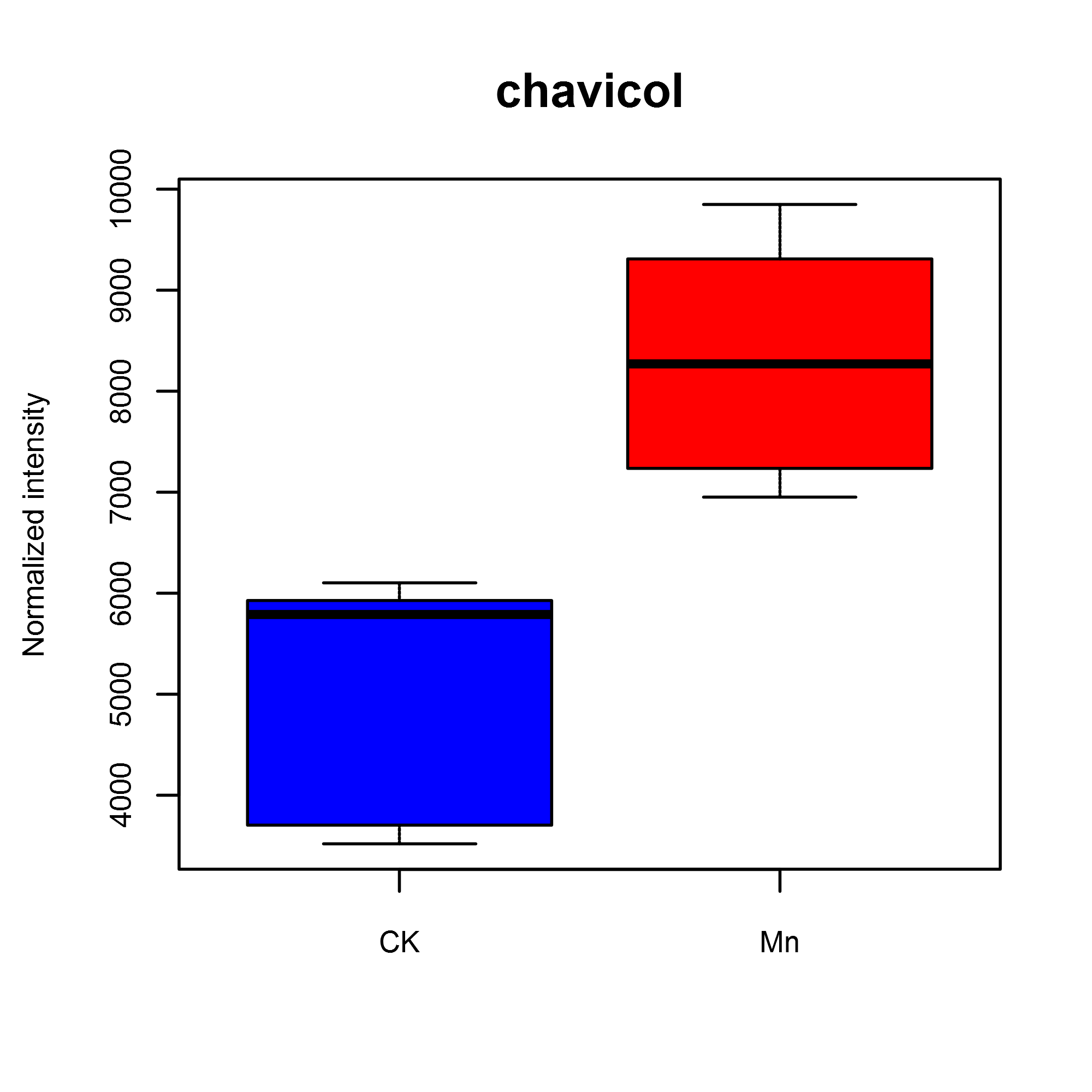

Supplement: Supplemental Information 3 — The raw data were for the LC-MS analysis including PLS-DA analysis in both positive and negative ionization mode, significantly differential metabolites of Ganoderma lucidum between treatments, mutual promotion or inhibition relationships between differential metabolites, etc. [file peerj-07-6846-s003.zip › raw data/CK vs Mn/visual/box/chavicol.png]

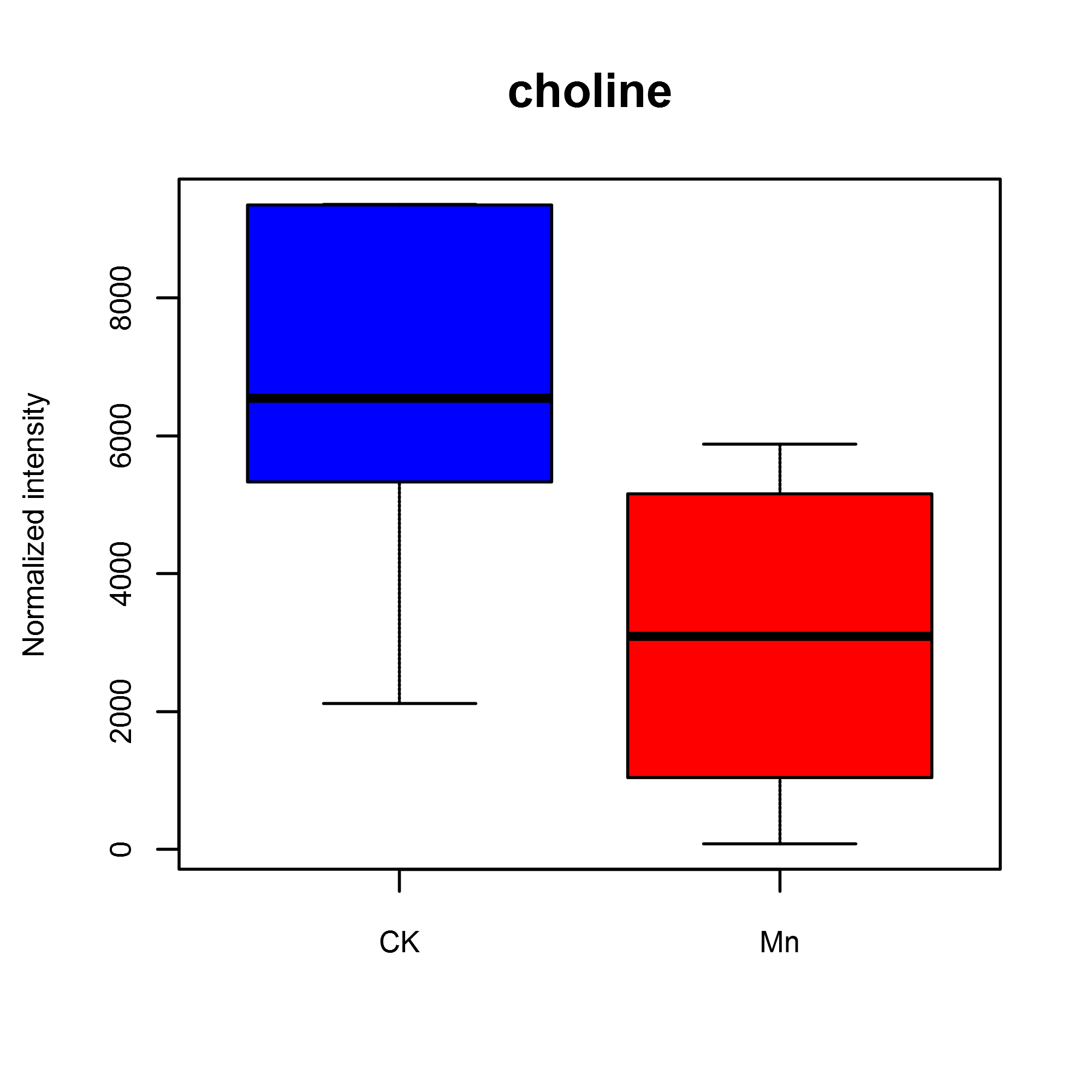

Supplement: Supplemental Information 3 — The raw data were for the LC-MS analysis including PLS-DA analysis in both positive and negative ionization mode, significantly differential metabolites of Ganoderma lucidum between treatments, mutual promotion or inhibition relationships between differential metabolites, etc. [file peerj-07-6846-s003.zip › raw data/CK vs Mn/visual/box/choline.png]

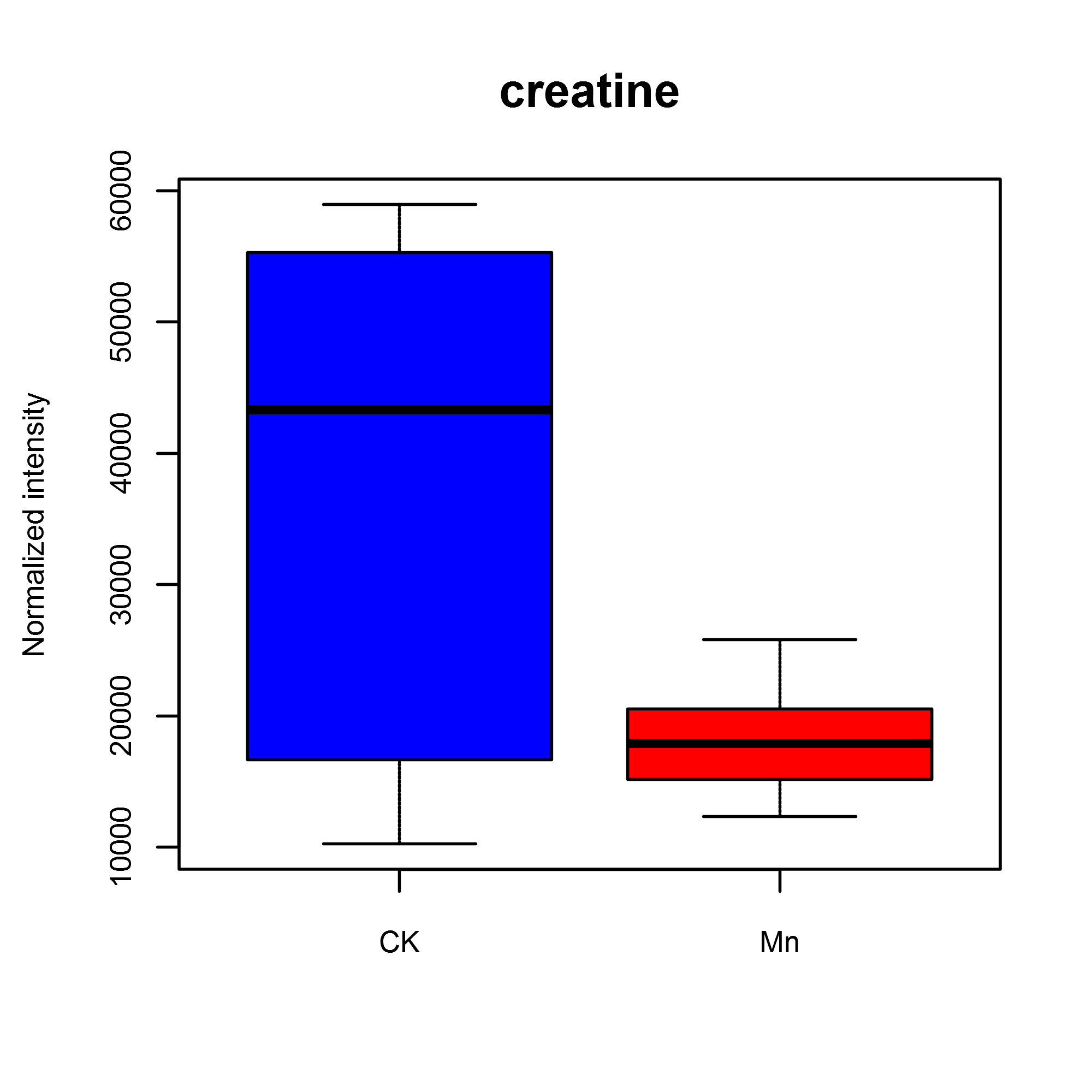

Supplement: Supplemental Information 3 — The raw data were for the LC-MS analysis including PLS-DA analysis in both positive and negative ionization mode, significantly differential metabolites of Ganoderma lucidum between treatments, mutual promotion or inhibition relationships between differential metabolites, etc. [file peerj-07-6846-s003.zip › raw data/CK vs Mn/visual/box/creatine.png]

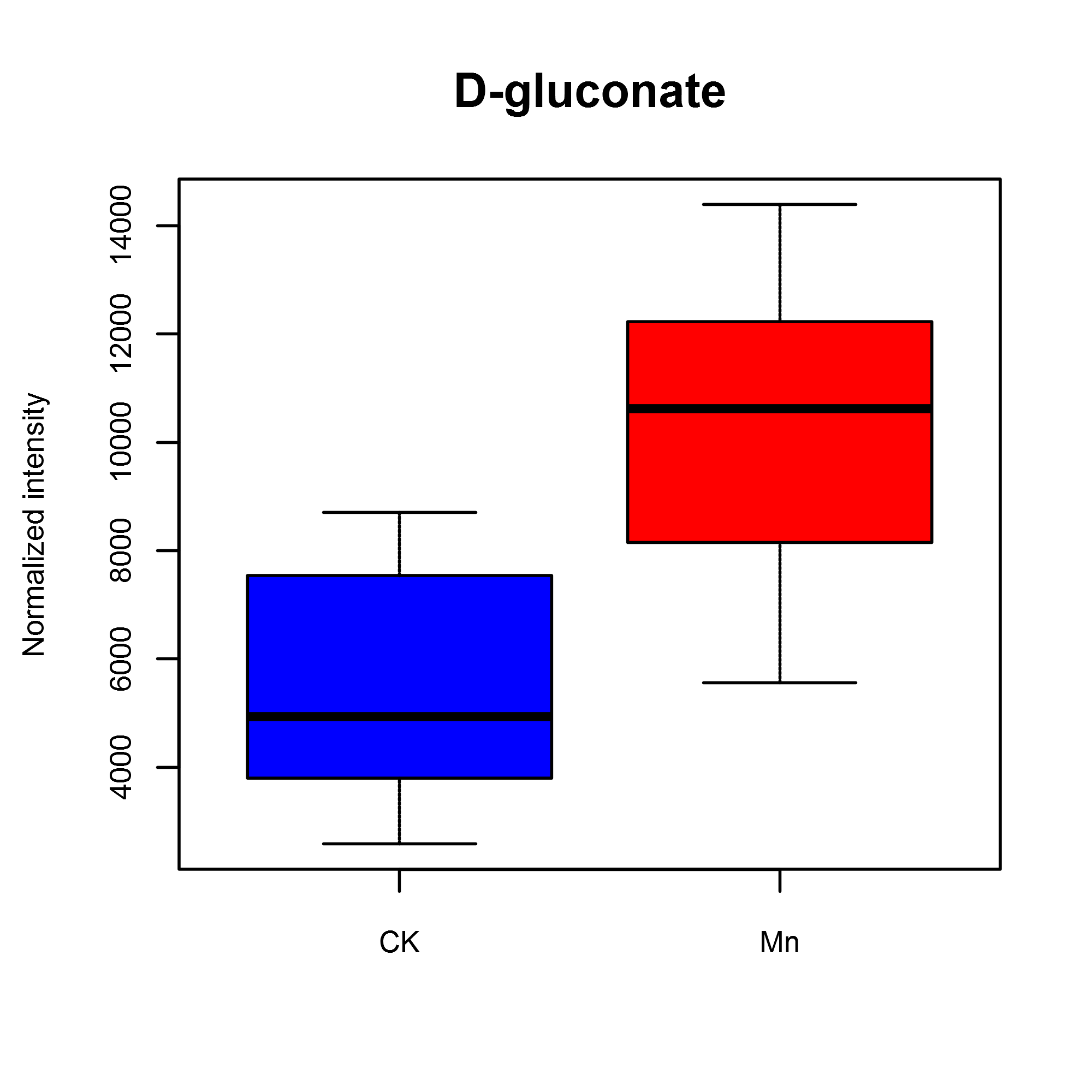

Supplement: Supplemental Information 3 — The raw data were for the LC-MS analysis including PLS-DA analysis in both positive and negative ionization mode, significantly differential metabolites of Ganoderma lucidum between treatments, mutual promotion or inhibition relationships between differential metabolites, etc. [file peerj-07-6846-s003.zip › raw data/CK vs Mn/visual/box/D-gluconate.png]

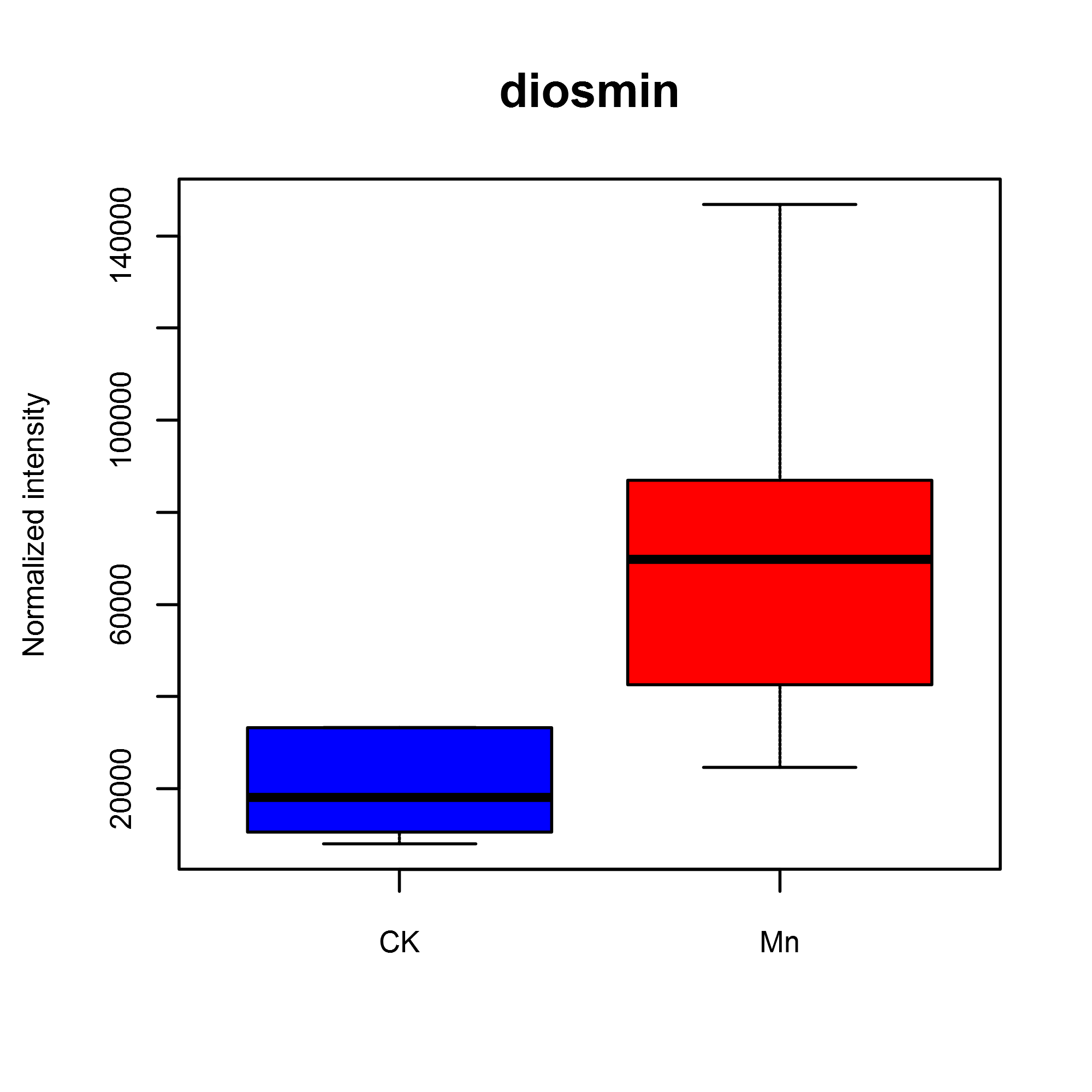

Supplement: Supplemental Information 3 — The raw data were for the LC-MS analysis including PLS-DA analysis in both positive and negative ionization mode, significantly differential metabolites of Ganoderma lucidum between treatments, mutual promotion or inhibition relationships between differential metabolites, etc. [file peerj-07-6846-s003.zip › raw data/CK vs Mn/visual/box/diosmin.png]

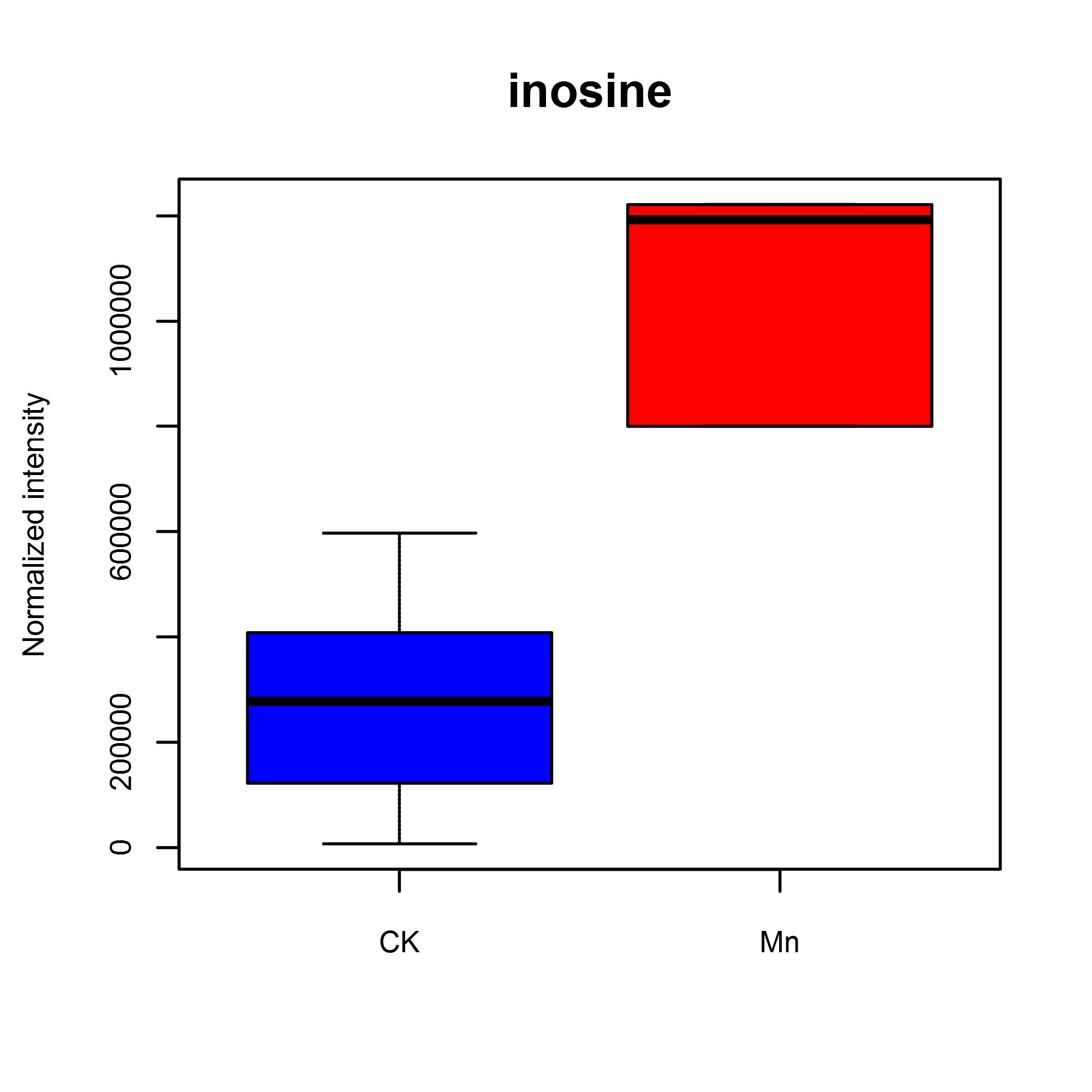

Supplement: Supplemental Information 3 — The raw data were for the LC-MS analysis including PLS-DA analysis in both positive and negative ionization mode, significantly differential metabolites of Ganoderma lucidum between treatments, mutual promotion or inhibition relationships between differential metabolites, etc. [file peerj-07-6846-s003.zip › raw data/CK vs Mn/visual/box/inosine.png]

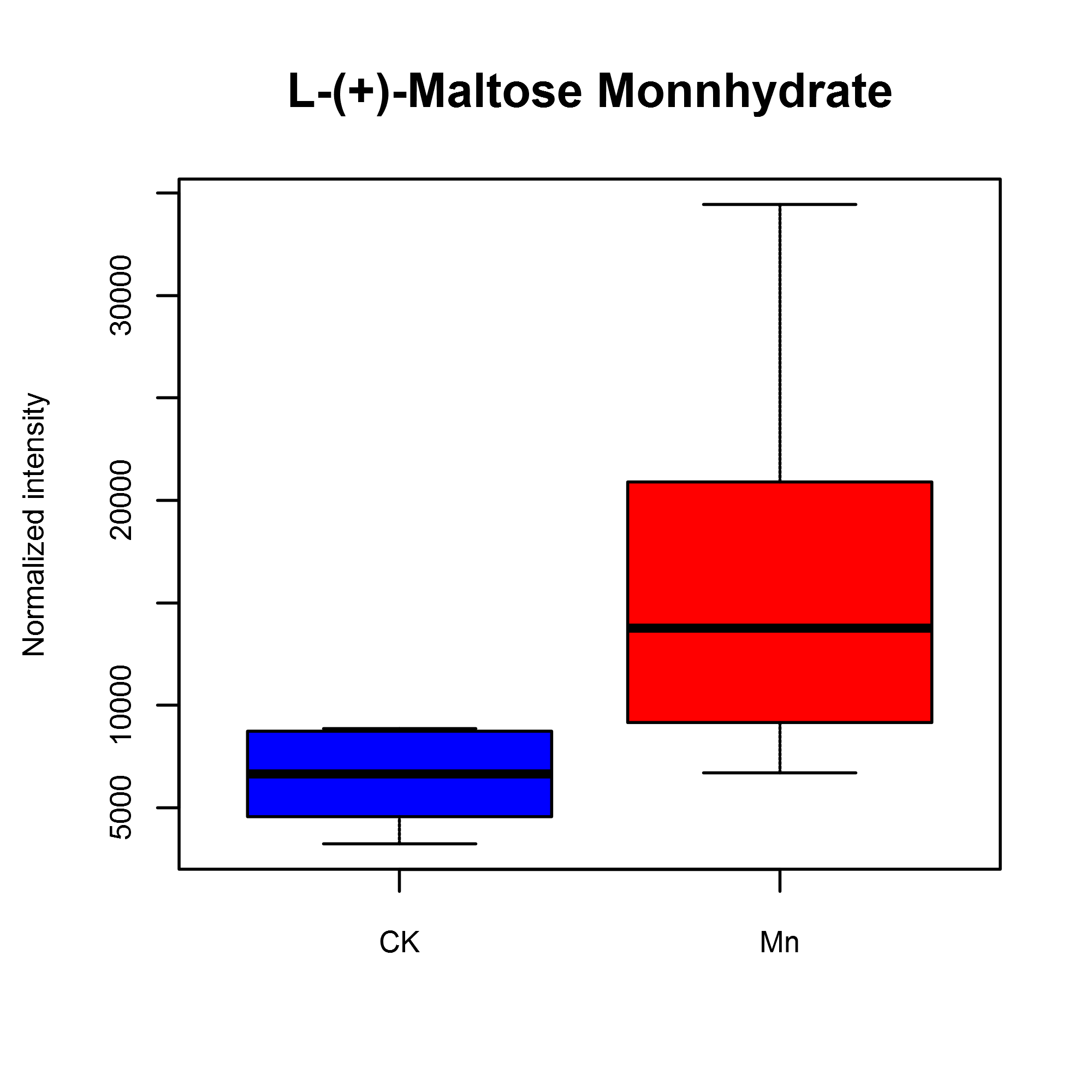

Supplement: Supplemental Information 3 — The raw data were for the LC-MS analysis including PLS-DA analysis in both positive and negative ionization mode, significantly differential metabolites of Ganoderma lucidum between treatments, mutual promotion or inhibition relationships between differential metabolites, etc. [file peerj-07-6846-s003.zip › raw data/CK vs Mn/visual/box/L-(+)-Maltose Monnhydrate.png]

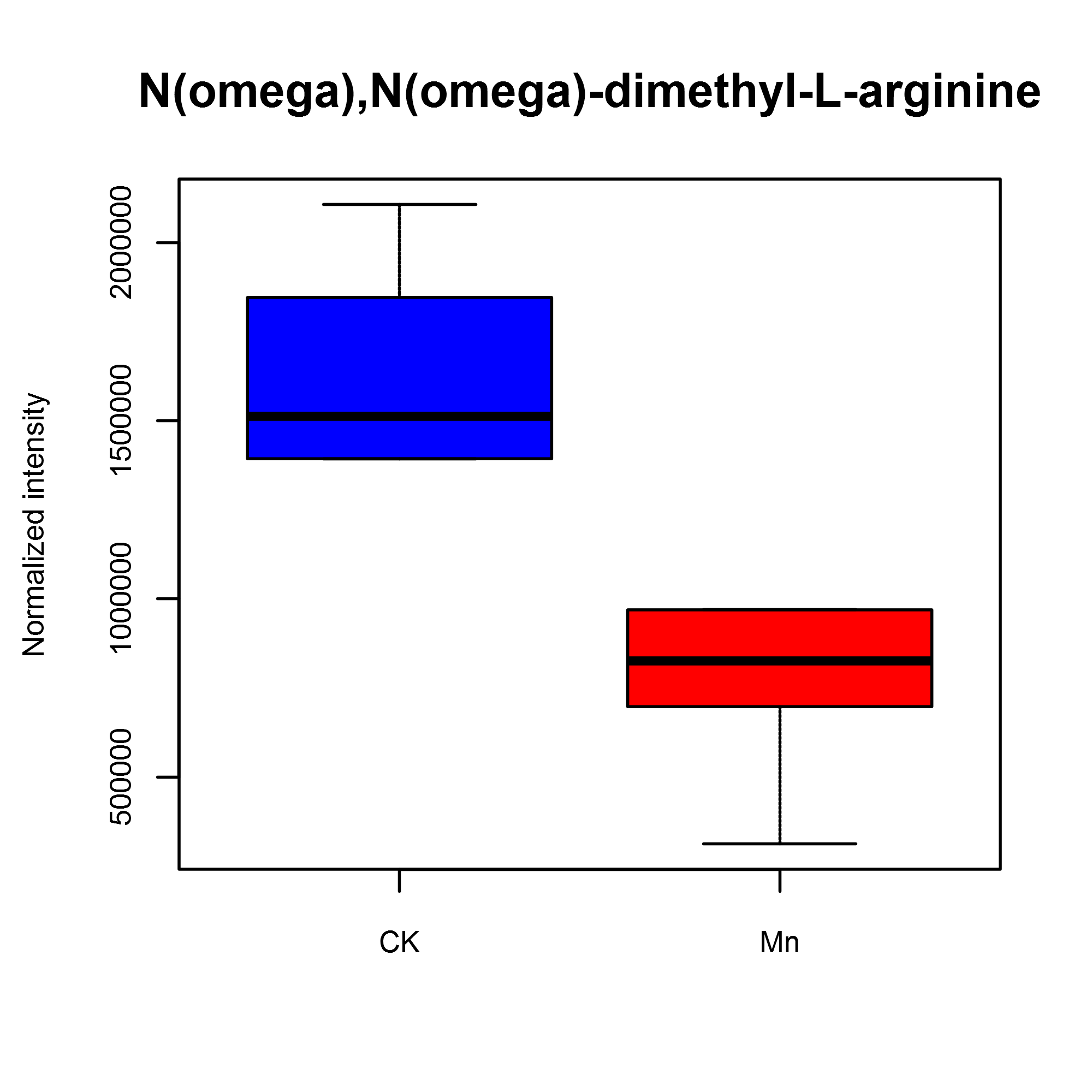

Supplement: Supplemental Information 3 — The raw data were for the LC-MS analysis including PLS-DA analysis in both positive and negative ionization mode, significantly differential metabolites of Ganoderma lucidum between treatments, mutual promotion or inhibition relationships between differential metabolites, etc. [file peerj-07-6846-s003.zip › raw data/CK vs Mn/visual/box/N(omega),N(omega)-dimethyl-L-arginine.png]

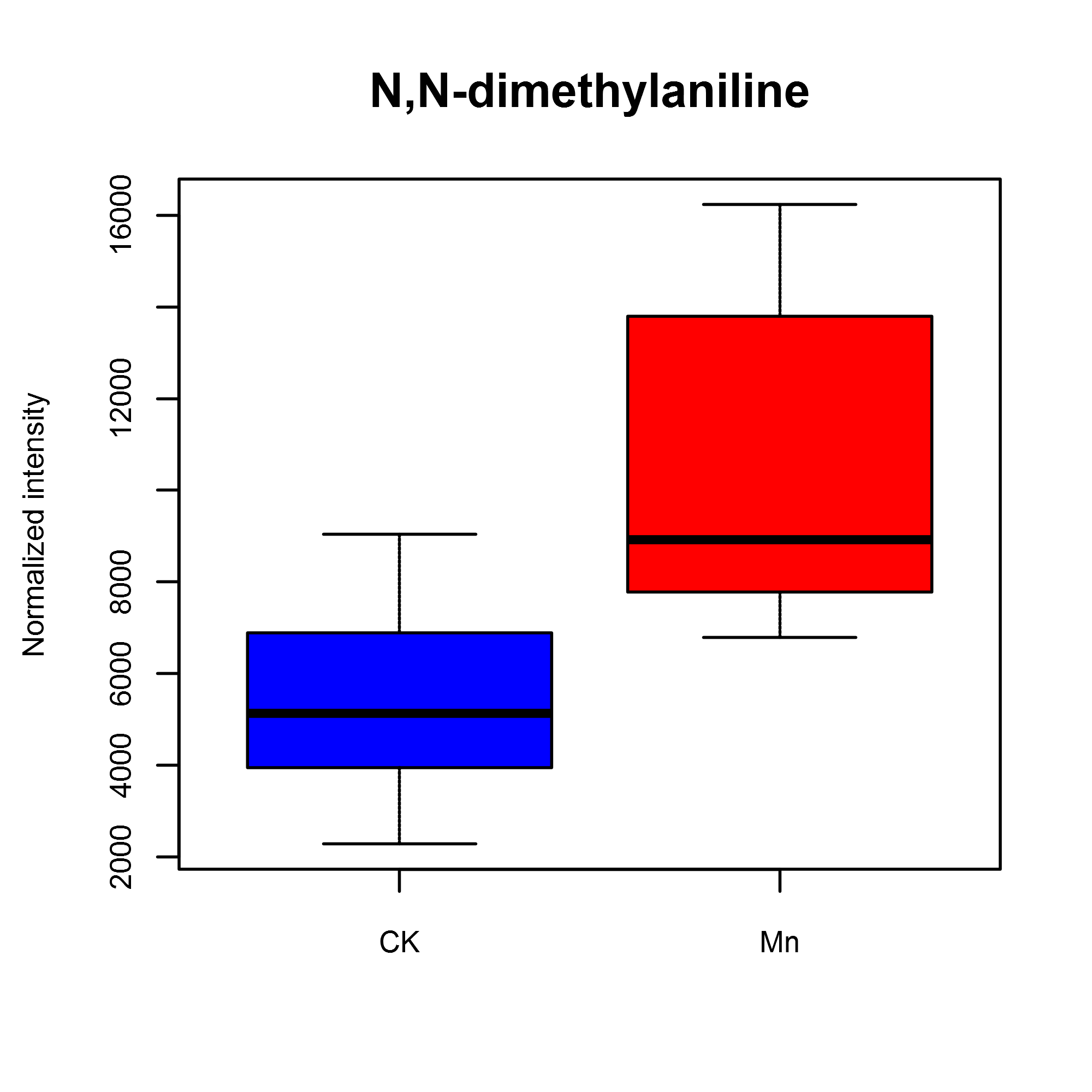

Supplement: Supplemental Information 3 — The raw data were for the LC-MS analysis including PLS-DA analysis in both positive and negative ionization mode, significantly differential metabolites of Ganoderma lucidum between treatments, mutual promotion or inhibition relationships between differential metabolites, etc. [file peerj-07-6846-s003.zip › raw data/CK vs Mn/visual/box/N,N-dimethylaniline.png]

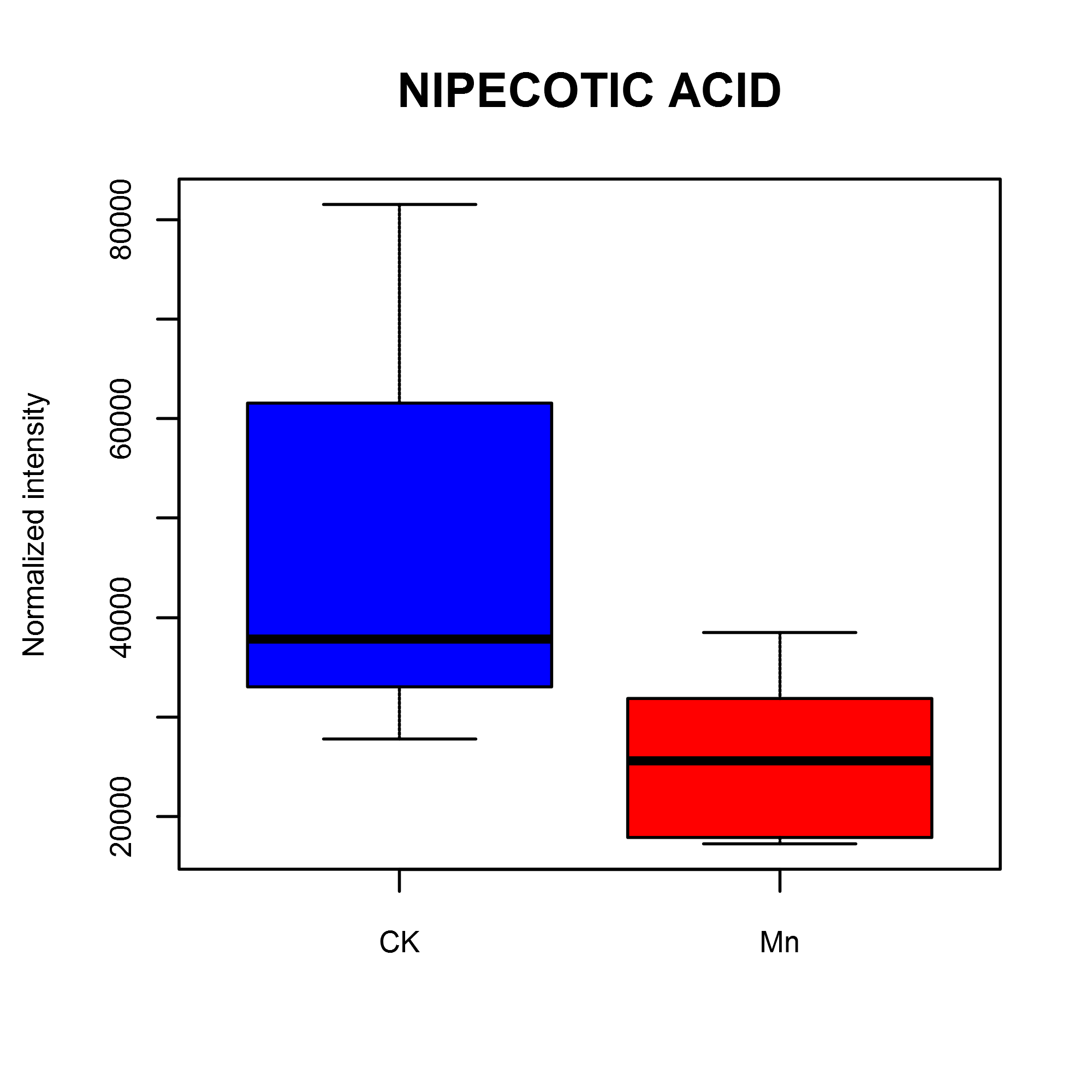

Supplement: Supplemental Information 3 — The raw data were for the LC-MS analysis including PLS-DA analysis in both positive and negative ionization mode, significantly differential metabolites of Ganoderma lucidum between treatments, mutual promotion or inhibition relationships between differential metabolites, etc. [file peerj-07-6846-s003.zip › raw data/CK vs Mn/visual/box/NIPECOTIC ACID.png]

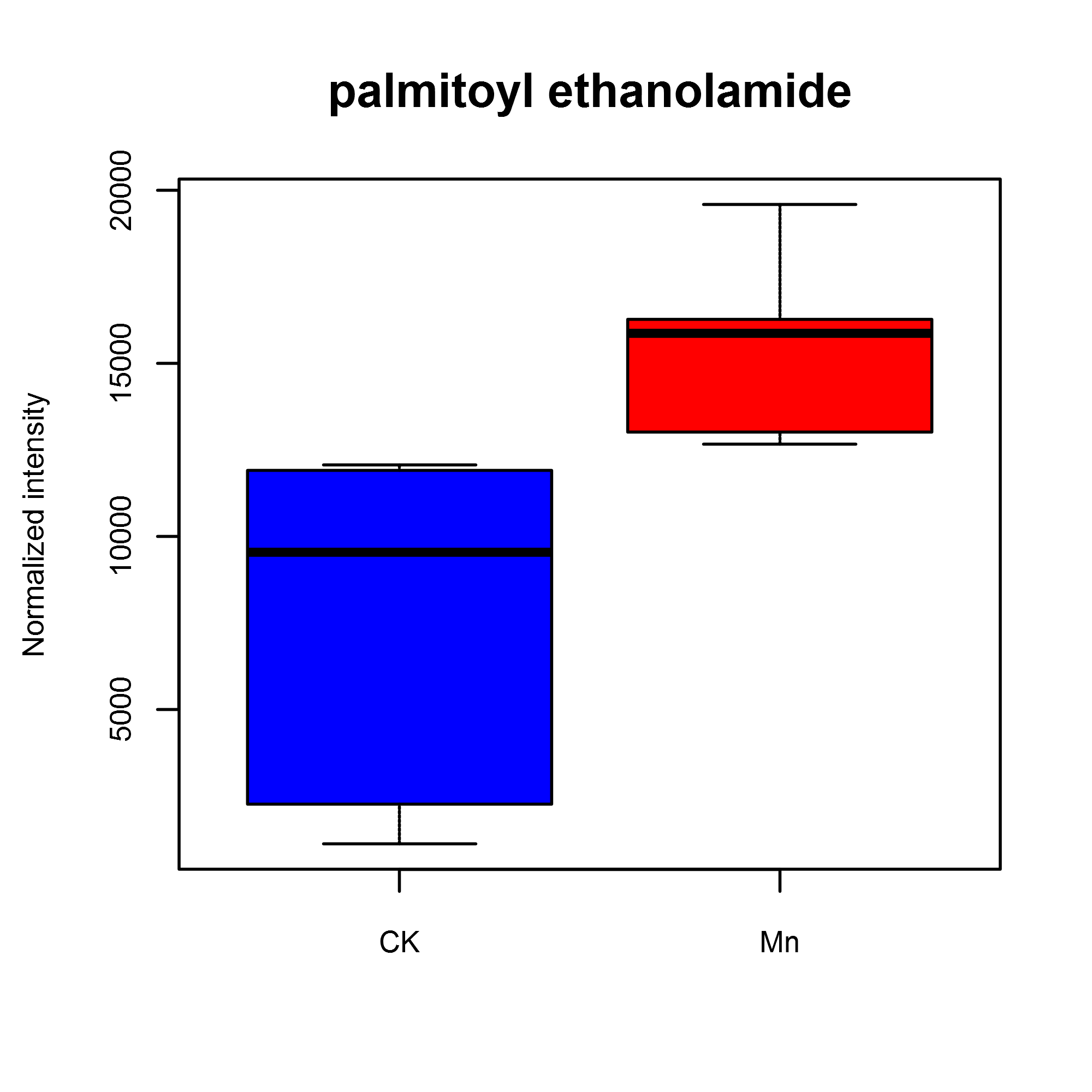

Supplement: Supplemental Information 3 — The raw data were for the LC-MS analysis including PLS-DA analysis in both positive and negative ionization mode, significantly differential metabolites of Ganoderma lucidum between treatments, mutual promotion or inhibition relationships between differential metabolites, etc. [file peerj-07-6846-s003.zip › raw data/CK vs Mn/visual/box/palmitoyl ethanolamide.png]
